# Infection with wild-type SARS-CoV-2 elicits broadly neutralizing and protective antibodies against omicron subvariants

**DOI:** 10.1038/s41590-023-01449-6

**Published:** 2023-03-13

**Authors:** Bin Ju, Qi Zhang, Ziyi Wang, Zhen Qin Aw, Peng Chen, Bing Zhou, Ruoke Wang, Xiangyang Ge, Qining Lv, Lin Cheng, Rui Zhang, Yi Hao Wong, Huixin Chen, Haiyan Wang, Sisi Shan, Xuejiao Liao, Xuanling Shi, Lei Liu, Justin Jang Hann Chu, Xinquan Wang, Zheng Zhang, Linqi Zhang

**Affiliations:** 1 Institute for Hepatology, National Clinical Research Center for Infectious Disease, Shenzhen Third People’s Hospital, Shenzhen, China; 2 The Second Affiliated Hospital, School of Medicine, Southern University of Science and Technology, Shenzhen, China; 3 Center for Global Health and Infectious Diseases, Comprehensive AIDS Research Center, Department of Basic Medical Sciences, School of Medicine, Tsinghua University, Beijing, China; 4 The Ministry of Education Key Laboratory of Protein Science, Beijing Advanced Innovation Center for Structural Biology, Beijing Frontier Research Center for Biological Structure, Collaborative Innovation Center for Biotherapy, School of Life Sciences, Tsinghua University, Beijing, China; 5 Biosafety Level 3 Core Facility, Yong Loo Lin School of Medicine, National University of Singapore, Singapore, Singapore; 6 Laboratory of Molecular RNA Virology and Antiviral Strategies, Department of Microbiology and Immunology, Yong Loo Lin School of Medicine, National University of Singapore, Singapore, Singapore; 7 Infectious Disease Translation Research Programme, Yong Loo Lin School of Medicine, National University of Singapore, Singapore, Singapore; 8 Guangdong Key Laboratory for Anti-infection Drug Quality Evaluation, Shenzhen, China; 9 Institute of Biopharmaceutical and Health Engineering, Tsinghua Shenzhen International Graduate School, Tsinghua University, Shenzhen, China; 10 Institute of Biomedical Health Technology and Engineering, Shenzhen Bay Laboratory, Shenzhen, China

**Keywords:** Immunology, Structural biology

## Abstract

The omicron variants of SARS-CoV-2 have substantial ability to escape infection- and vaccine-elicited antibody immunity. Here, we investigated the extent of such escape in nine convalescent patients infected with the wild-type SARS-CoV-2 during the first wave of the pandemic. Among the total of 476 monoclonal antibodies (mAbs) isolated from peripheral memory B cells, we identified seven mAbs with broad neutralizing activity to all variants tested, including various omicron subvariants. Biochemical and structural analysis indicated the majority of these mAbs bound to the receptor-binding domain, mimicked the receptor ACE2 and were able to accommodate or inadvertently improve recognition of omicron substitutions. Passive delivery of representative antibodies protected K18-hACE2 mice from infection with omicron and beta SARS-CoV-2. A deeper understanding of how the memory B cells that produce these antibodies could be selectively boosted or recalled can augment antibody immunity against SARS-CoV-2 variants.

## Abstract (web summary)

Zhang and colleagues perform functional, biochemical and structural analysis of a set of antibodies isolated from COVID-19 convalescents infected with wild-type SARS-CoV-2 during the first wave of the pandemic and show they have broad neutralizing activity against all SARS-CoV-2 variants tested, including omicron.

## Main

The rapid emergence and turnover of SARS-CoV-2 variants of concern (VOCs) and progressive waning of antibody immunity have raised serious concerns that the current vaccine strategies based on the wild-type spike (S) protein would not be able to provide sufficient protection against the antigenically distinct VOCs, particularly in the case of omicron^1–4^. The omicron subvariants are the most efficient in transmission and have not only been associated with steeply increased new infections among unvaccinated individuals, but also with break-through infections among those vaccinated^5–7^. The exceptionally high number of mutations in the S protein of omicron has resulted in considerable antigenic shift from the wild-type and previously identified VOCs^8–11^ and renders strong capability to escape from neutralization of convalescent, vaccinated and hybrid immunized (convalescent/vaccinated or vaccinated/convalescent) individuals^3,4,8,12^. However, one of the major questions that remains to be answered is the extent of such escape and whether antibodies in the infected or vaccinated individuals can neutralize the VOCs identified so far. If such neutralizing antibodies (nAbs) do exist, it is important to identify their specific features and whether they could be selectively boosted by vaccines to confer broader and more sufficient protection against VOCs, particularly omicron subvariants.

The current work aimed to dissect the antibody repertoire of nine convalescent patients infected with the wild-type SARS-CoV-2, enrolled during the first wave of the COVID-19 pandemic. The naïve background of these individuals allowed us to evaluate the antibody imprint of the wild-type SARS-CoV-2 infection, as well as the immunological potential of these antibodies against subsequently emerged VOCs. We isolated and characterized a total of 476 mAbs from the peripheral memory B cells 16–111 d post symptom onset, and identified seven receptor-binding domain (RBD)-specific mAbs able to neutralize all variants tested, including omicron subvariants. Passive delivery of four representative antibodies of the broad nAbs (bnAbs) protected K18-hACE2 mice from infection with omicron or beta. Biochemical, structural and functional characterization of these bnAbs revealed unique as well as common features associated with their neutralizing breadth and potency.

## Results

### S2-binding mAbs dominate in B cell receptor repertoires

**Fig. 1:**
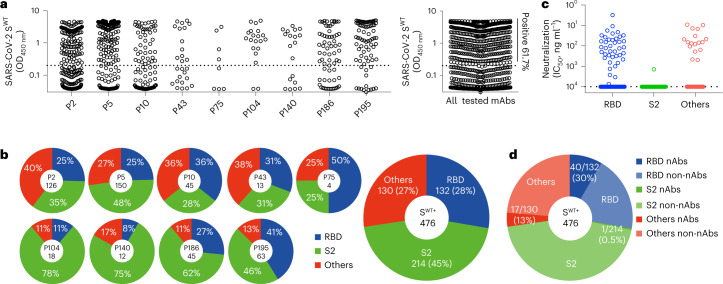
Identification and characterization of S^WT^-specific antibodies from convalescent patients in the early pandemic. **a**, Initial screening for S^WT^-specific antibodies from nine convalescent patients using ELISA. Of 771 antibodies produced in the culture supernatant, 476 (61.7%) had strong binding with optical density (OD) values above the cut-off of 0.2, more than threefold higher than the background. **b**, Epitope specificity of the 476 antibodies identified in **a**. The patient number and the number of antibodies obtained from each patient are shown in the inner circle. Each slice is colored according to its epitope specificity and is proportional to the total antibodies obtained. **c**, Initial screening of the 476 antibodies as in **a** for their neutralizing activity against wild-type SARS-CoV-2 using pseudovirus-based neutralization assay. The cut-off value of neutralization was 10,000 ng ml^−1^. **d**, Proportions of nAbs among those binding to RBD (40 of 132, 30%), S2 (1 of 214, 0.5%) and others (17 of 130, 13%). Results are representative of at least two independent experiments. IC_50_, half-maximum inhibitory concentration. Source data

**Extended Data Fig. 1:**
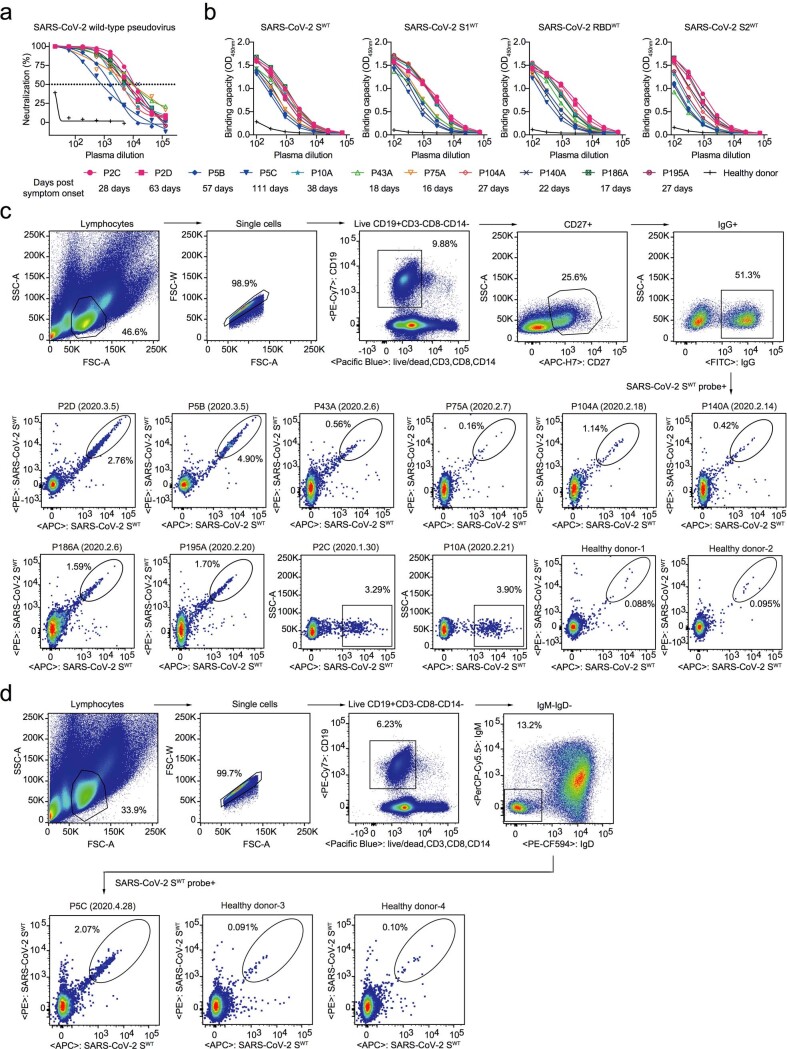
Analysis of plasma antibody response and S^WT^-specific single B cells from convalescent patients. **a, b**, Neutralizing **(a)** and binding **(b)** activity of convalescent plasma analyzed by wild-type pseudovirus and ELISA. Recombinant S, S1, RBD, and S2 proteins derived from the wild-type strain was used in the ELISA. The samples are labeled as A, B, C, or D depending on the collection sequence. The date of sampling was presented as days post symptom onset. Results calculated from two independent experiments. **c-d**, Gating strategy for isolating S^WT^-specific single B cells from 9 convalescent individuals by FACS. PBMCs from four healthy donors were used as the background control. S^WT^-specific single B cells were either gated as **(c)** CD19^+^CD3^−^CD8^−^CD14^−^CD27^+^IgG^+^S^WT+^ or **(d)** CD19^+^CD3^−^CD8^−^CD14^−^IgM^−^IgD^−^S^WT+^. The percentage of S^WT^-specific single B cells from each sample were shown. FSC-A: forward scatter area. SSC-A: side scatter area. FSC-W: forward scatter width. Source data

**Extended Data Fig. 2:**
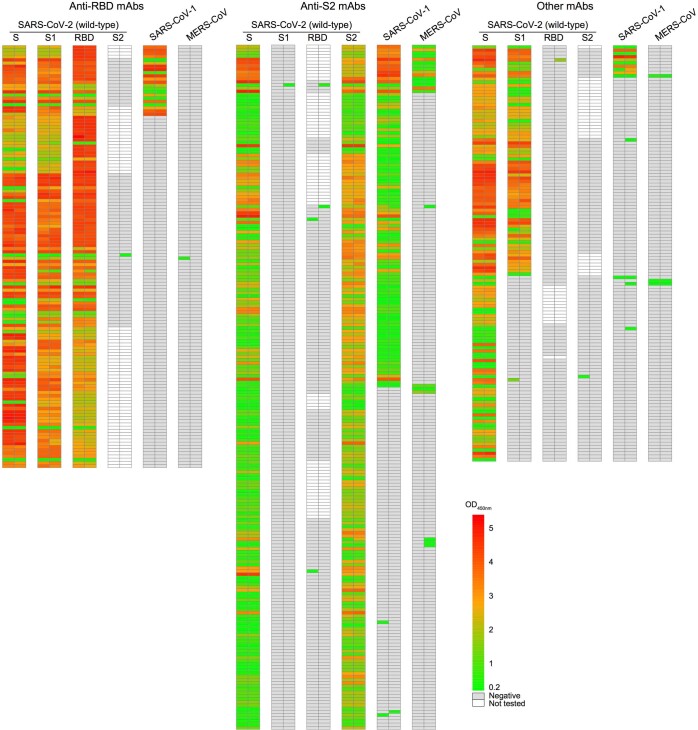
Binding specificity of 476 isolated antibodies. Binding specificity was analyzed by ELISA against recombinant S, S1, RBD, and S2 proteins of wild-type SARS-CoV-2, and S1 or S proteins of SARS-CoV-1 and MERS-CoV. Each antibody was tested in duplicates and presented as such on the same row. The binding activity is colored from red to green, with red being the strongest and green just above the optical density (OD) cut-off value of 0.2, at least 3−fold higher than the background. The negative ones are in light grey with OD_450nm_ < 0.2. Source data

Using peripheral blood mononuclear cells (PBMCs) from nine convalescent individuals (P2, P5, P10, P43, P75, P104, P140, P186 and P195) collected during the early wave of the pandemic between January and April of 2020 (Supplementary Table 1), when only the wild-type SARS-CoV-2 was circulating^13^, we analyzed the memory B cell receptor repertoires specific to the wild-type S (S^WT^) trimer (Extended Data Fig. 1). We were able to obtain a total of 771 paired antibody heavy and light variable genes from flow cytometry-sorted single B cells. Once expressed in HEK 293T cells, 476 of the 771 antibodies bound to the recombinant S^WT^ trimer (Fig. 1a). The number of such high-binding mAbs varied from patient to patient (Fig. 1b), perhaps due to variability in cell conditions and time points when samples were collected. Among the 476 mAbs, 214 (45%) were S2-specific, 132 (28%) were RBD-specific and 130 (27%) were either NTD-specific or targeting the quaternary epitopes that only exist in the form of S^WT^ trimer^14^ (Fig. 1b and Extended Data Fig. 2). S2-specific mAbs tended to cross-react with the recombinant S trimer from SARS-CoV-1 and with only a few from MERS-CoV (Extended Data Fig. 2). In contrast, RBD-specific and the remaining mAbs were largely SARS-CoV-2-specific, with only a few cross-reacting with SARS-CoV-1 and even fewer with MERS-CoV (Extended Data Fig. 2). Thus, S2-binding mAbs dominated among the isolated 476 mAbs specific to the SARS-CoV-2 S^WT^ trimer.

### Broad and potent nAbs neutralize various SARS-CoV-2 variants

**Fig. 2:**
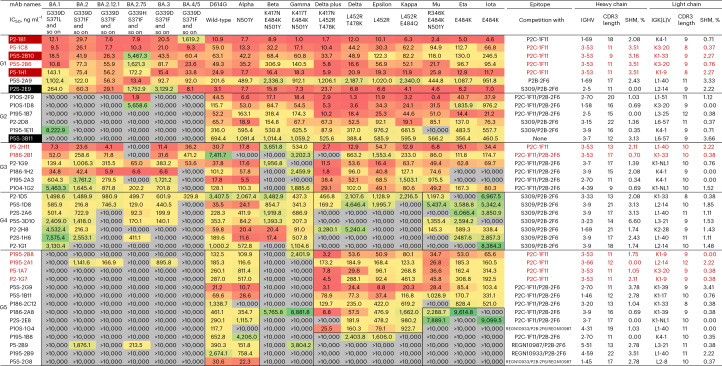
Neutralizing activity, epitope characterization and gene family analysis of the top 40 RBD-specific antibodies. Neutralization activity against a panel of 17 pseudoviruses carrying the S proteins of wild-type and various VOCs and VOIs, indicated by antibody concentration required to achieve 50% reduction in viral infectivity (IC_50_, ng ml^−1^). The neutralizing activity is colored from red, orange, yellow, green to gray, with red being the strongest, while gray failed to reach IC_50_ at the highest concentration tested (10,000 ng ml^−1^). A few representative mutations that potentially facilitate viral escape from antibodies are indicated below each of the VOCs and VOIs. The complete set of mutations for each of the VOCs and VOIs can be found in the Methods. Epitope specificity was determined by competition with typical class 1 (P2C-1F11 and REGN10933), class 2 (P2B-2F6) and class 3 (REGN10987 and S309) antibodies measured by surface plasmon resonance (SPR). All results were calculated from at least two independent experiments. The germline gene usage (IGHV, IGKV, IGLV), the length of complementarity determining region (CDR) 3 and the proportion of somatic hypermutation (SHM) were estimated using the IMGT program. Antibodies highlighted in red text are those competed with typical class 1 antibody P2C-1F11 and which preferentially used germline IGHV3-53/66. For clarity, the five antibodies that had their crystal or cryo-EM structures resolved in complex with RBD or S trimer are labeled with either red (class 1) or black (other classes) background. G1 to G5, group 1 to group 5; VOIs, variants of interest.

**Extended Data Fig. 3:**
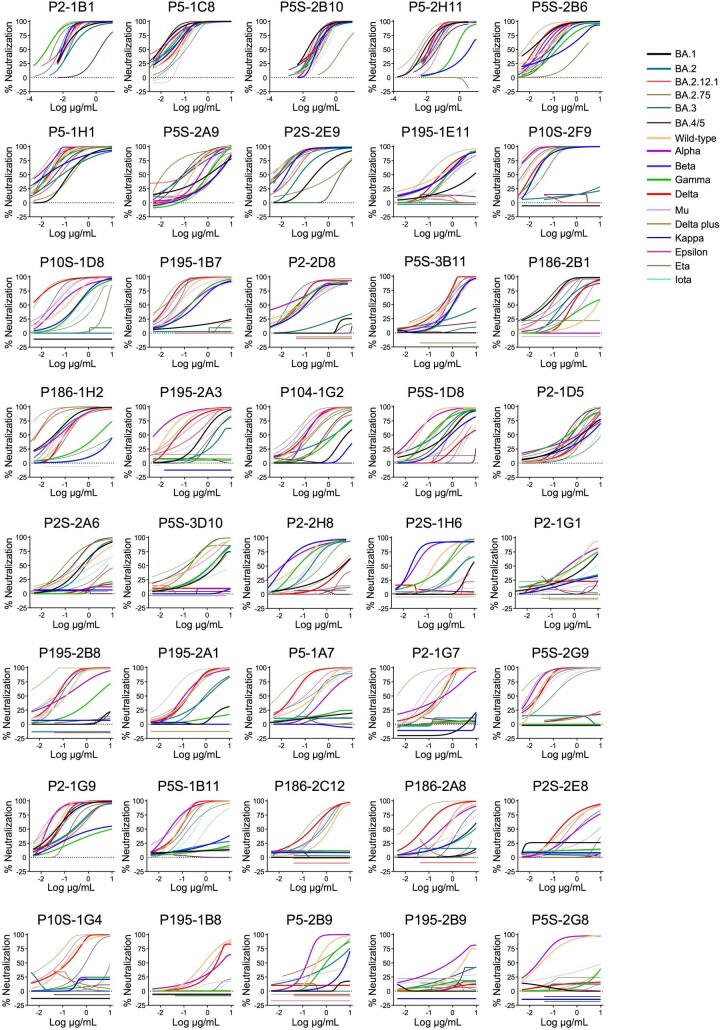
Neutralization activity of top 40 RBD-specific antibodies against pseudoviruses bearing spike of wild-type, major VOCs and other variants of SARS-CoV-2. Pseudoviruses were tested against serial dilutions of the testing antibodies. Neutralization activity was defined as the concentration required to achieve 50% reduction in viral infection (IC_50_) relative to no antibody controls. Results were calculated from at least two independent experiments. One representative curve was displayed. Source data

**Extended Data Fig. 4:**
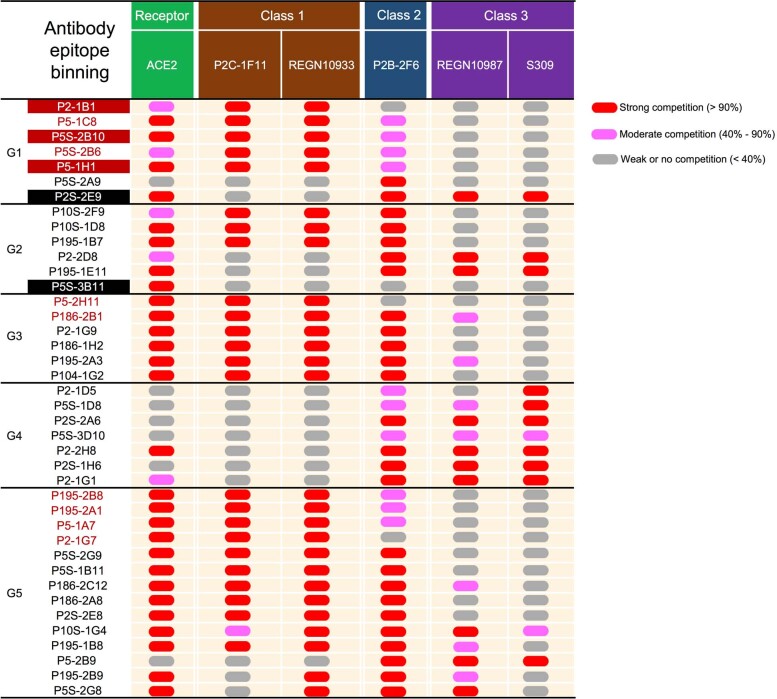
Epitope characterization of top 40 RBD-specific antibodies. Epitope specificity was determined by competition with ACE2, typical class 1 (P2C-1F11 and REGN10933), class 2 (P2B-2F6), and class 3 (REGN10987 and S309) antibodies measured by surface plasmon resonance (SPR). For clarity, the five antibodies had their crystal or cryo-EM structures resolved in complex with RBD or S trimer are highlighted in either red (class 1) or back (other classes) background. G1 to G5: group 1 to group 5.

We next found that approximately 30% (40 of 132) of RBD-specific mAbs demonstrated neutralizing activity against wild-type SARS-CoV-2 pseudovirus, whereas this was detected for only 13% (17 of 130) of the other mAbs and less than 1% (1 of 214) of the S2-specific antibodies (Fig. 1c,d). Furthermore, through screening against a panel of 17 pseudoviruses bearing the S protein from the VOCs, including omicron subvariants and other variants under global surveillance, we were able to categorize the top 40 RBD-specific mAbs into five major groups based on their distinctive neutralizing breadth and potency (Fig. 2 and Extended Data Fig. 3). Group 1 included seven mAbs (P2-1B1, P5-1C8, P5S-2B10, P5S-2B6, P5-1H1, P5S-2A9 and P2S-2E9) capable of neutralizing the entire panel of pseudoviruses, despite some having a marked reduction in neutralization against omicron (Fig. 2). Among the seven group 1 mAbs, P5-1C8 demonstrated an extremely broad and potent neutralizing activity across all variants tested, while the rest of the mAbs were somewhat compromised in their neutralizing activity against omicron BA.1 (P5-1H1, P5S-2A9 and P2S-2E9), BA.2.75 (P5S-2B10 and P5S-2B6) or BA.4/5 (P2-1B1). Reduced neutralization against beta and gamma (P5S-2B6 and P5S-2A9) was perhaps due to the K417N/T, E484K and N501Y mutations^1,15^, while that against omicron subvariants was likely due to the constellation substitutions found in the RBD, such as S371L/F, S375F, N440K, G446S, N460K, E484A, F486V, Q493R, Q498R and N501Y. Epitope mapping through competition with five mAbs of known structure and epitope specificity (P2C-1F11, P2B-2F6, S309, REGN10933 and REGN10987) indicated five of the seven antibodies (P2-1B1, P5-1C8, P5S-2B10, P5S-2B6 and P5-1H1) strongly competed with P2C-1F11 (Fig. 2 and Extended Data Fig. 4), a typical class 1 antibody that binds to RBD through ACE2 mimicry. Four antibodies in group 1 (P5-1C8, P5S-2B10, P5S-2B6 and P5-1H1) used the immunoglobulin G heavy-chain variable (IGHV) germline region IGHV3-53, a disproportionately favored germline gene found in over 10% of RBD-specific nAbs^16^. The remaining two mAbs in group 1 (P5S-2A9 and P2S-2E9) competed either with P2B-2F6, a typical class 2 antibody, or with P2B-2F6 and S309, a typical class 3 antibody (Fig. 2 and Extended Data Fig. 4), suggesting they bound to the outer face of the RBD. All seven mAbs in group 1 contained minimal somatic mutations (0.00–3.51%) and had standard HCDR3 length (9–18 residues), which aligned well with previous reports^17^. These results indicate that infection with wild-type SARS-CoV-2 could generate broad and potent neutralizing mAbs that targeted multiple epitopes on RBD, although mimicking ACE2 binding to RBD with preferred usage of germline gene IGHV3-53 heavily dominated.

Group 2 to group 5 antibodies appeared to be specifically affected by the different VOCs and variants tested (Fig. 2). Before the emergence of omicron variants, group 2 antibodies had impressive neutralizing breadth and potency across all variants tested (Fig. 2). However, widespread mutations found on the RBD of omicron have facilitated escape from the neutralizing activity of this group of antibodies (Fig. 2). One antibody, P5S-3B11, failed to compete with any of the five representative mAbs used (Fig. 2), indicating its epitope was beyond the regions these antibodies recognized. Group 3 and group 4 antibodies, while maintaining some activity against omicron, were essentially uniform in competing with known antibodies (Fig. 2). Group 3 antibodies competed with P2C-1F11 and P2B-2F6, whereas group 4 antibodies competed with S309 and P2B-2F6 (Fig. 2). Such uniformity indicated the epitopes of group 3 and group 4 antibodies were rather focused, as opposed to those widely distributed found in group 2 antibodies (Fig. 2). As a result, a few mutations, such as K417N/T, E484K and N501Y found in beta and gamma and L452R and/or E484Q in delta plus, delta, epsilon and kappa, respectively, had led to severe reduction or complete loss of neutralization in group 3 and 4 antibodies. Two antibodies in group 3 (P186-2B1 and P186-1H2) and one in group 4 (P2-1D5) improved their neutralizing activity to some omicron subvariants relative to wild-type (D614G). Finally, group 5 antibodies were most vulnerable to the mutations found in VOCs and the other variants. While the majority of group 5 antibodies maintained neutralizing activity to the wild-type (D614G) and alpha, almost all of them failed to neutralize omicron subvariants, beta and gamma (Fig. 2). Close to 29% (4 of 14) and 50% (7 of 14) of group 5 antibodies lost their neutralizing activity to L452R- and E484K-containing variants, respectively (Fig. 2). Epitope mapping through competition also indicated the epitope distributions of group 5 antibodies were rather broad and diverse on the RBD (Fig. 2). Taken together, these results indicated that there was temporal and progressive loss of nAbs in convalescent patients as the new VOCs and variants continued to emerge and circulate in the population; however, antibodies such as those in group 1, which constituted about 18% (7 of 40) of the top RBD nAbs and 1.5% (7 of 476) of total S protein-binding antibodies, maintained broad and potent neutralizing activity.

### bnAbs disproportionally favor IGHV3-53 germline

**Fig. 3:**
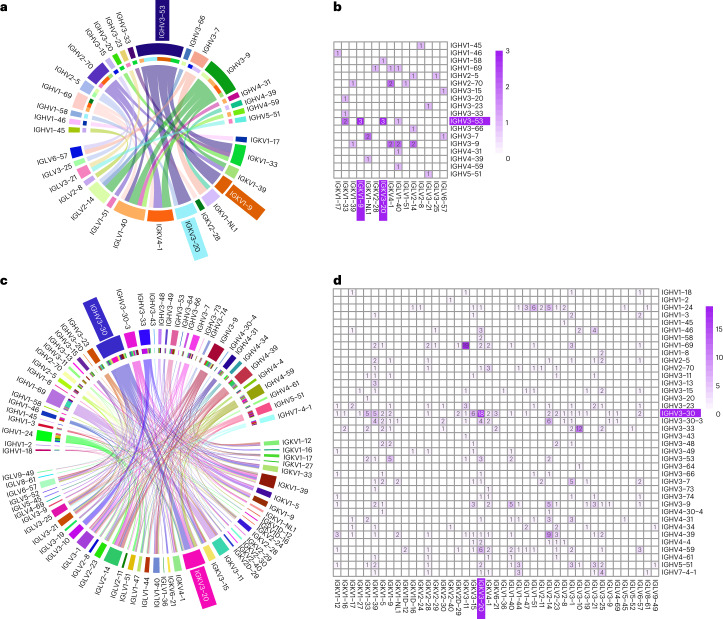
Preferred germline gene usage among the RBD-specific and the S-specific antibodies. **a**–**d**, Germline heavy and light gene usage and pairing among the top 40 RBD-specific (**a**,**b**) and the 476 S-specific antibodies (**c**,**d**), presented in chord diagrams (**a**,**c**) and heatmaps (**b**,**d**). In the chord diagrams, each of the paired germline heavy and light chains are linked by arcs, the sizes of which are proportional to the total antibodies identified. The number and the color in the heatmaps represent the number of antibodies identified with their germline heavy and light chains indicated along the vertical and horizontal axes. The preferred germline usages are highlighted by colored background in both chord diagrams and heatmaps. Source data

To characterize the genetic features of the five groups of RBD-specific nAbs, we analyzed their heavy and light chain repertories using the IMGT/V-QUEST program (http://www.imgt.org/IMGT_vquest/vquest). Similar to previous reports from naturally infected and vaccinated individuals^16,18,19^, IGHV3-53/66 were overrepresented among the total of 18 heavy chain families (25%; 10 of 40), whereas IGKV4-1 dominated in 8 kappa families (13%; 5 of 40) and IGLV1-40 dominated in 7 lambda families (15%; 6 of 40) (Fig. 3a,b and Supplementary Table 2). A preferred pairing between IGHV3-53/66 and IGKV1-9 and IGKV3-20 was identified and reached as high as 30% each among the total IGHV3-53/66 (Fig. 3b). When similar analysis was expanded to study all 476 S^WT^ trimer-binding antibodies, dominance was found for germline gene IGHV3-30 (13%) among the total of 38 heavy chain families, IGKV3-20 (11%) among 22 kappa families and IGLV2-14 (8%) among 21 lambda families (Fig. 3c). Preferential pairing between IGHV3-30 and IGKV3-20 was found and reached as high as 28% among the IGHV3-30 family members (Fig. 3d), which were all S2-specific (Fig. 1b and Extended Data Fig. 2). Therefore, neutralizing and binding antibodies tended to target different regions of the S protein with rather distinct germline gene preference for heavy and light chains as well as a combination thereof.

### bnAbs protect K18-hACE2 mice against omicron and beta

We next studied the protective potential of four antibodies in group 1 (P2-1B1, P5S-2B10, P5-1H1 and P2S-2E9) against infection with omicron BA.1 or beta variant in a model of SARS-CoV-2 infection in K18-hACE2 mice. In mice infected with omicron BA.1, 12 mice per group were intraperitoneally administered with P2-1B1, P5S-2B10 or P5-1H1 at a dose of 10 mg per kg body weight. In untreated controls, 12 mice were included for P2-1B1 whereas another 12 were shared by P5S-2B10 and P5-1H1. In mice infected with the beta variant, eight mice each received P2S-2E9 intraperitoneally at a dose of 10 mg per kg body weight or remained untreated. At 24 h later, all mice were intranasally challenged with 1.7 × 10^3^ plaque-forming units (PFU) of omicron BA.1 or beta variant. Half of the mice in each group were monitored daily for body weight and survival for 14 d. The remaining half were analyzed for viral titers in the lungs and brain and underwent histopathological analysis on day 3 (omicron BA.1) or day 4 (beta) post infection.

**Fig. 4:**
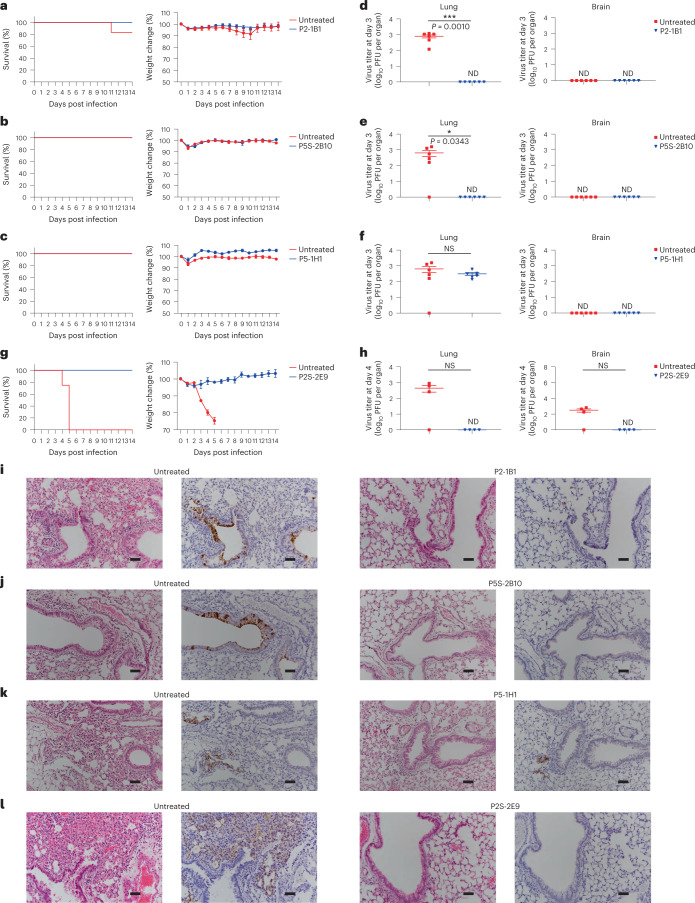
P2-1B1, P5S-2B10, P5-1H1 and P2S-2E9 prophylaxis protects K18-hACE2 mice from infection with SARS-CoV-2 omicron BA.1 or beta. **a**–**c**, Survival percentage and body weight recorded daily post infection with BA.1 until death occurred or at the end point of experiments at 14 dpi in P2-1B1 (**a**), P5S-2B10 (**b**) and P5-1H1 (**c**) groups. **d**–**f**, Lung viral titers and brain viral titers in mice killed at 3 dpi for BA.1 in P2-1B1 (**d**), P5S-2B10 (**e**) and P5-1H1 (**f**) groups. **g**, Survival percentage and body weight recorded daily post infection with beta until death occurred or at the end point of experiments at 14 dpi in P2S-2E9 group. **h**, Lung viral titers and brain viral titers in mice killed at 4 dpi for beta in P2S-2E9 group. Weight change and PFU per organ are presented as mean ± s.e.m. The significance was estimated by a two-tailed, unpaired *t-*test. *P < 0.05; ***P < 0.001; NS, not significant; ND, not detected. **i**–**l**, H&E and immunohistochemistry staining of lung tissue from P2-1B1 (**i**), P5S-2B10 (**j**), P5-1H1 (**k**) or P2S-2E9 (**l**) intraperitoneally treated and corresponding untreated mice at day 3 (BA.1) or day 4 (beta) post infection. Dark brown, cells positive for SARS-CoV-2 N protein. Scale bars, 50 µm. Images were derived from one representative mouse in each group. The P2-1B1 experiment shared the same negative control mice as previously published^50^. P5S-2B10 and P5-1H1 experiments shared the same negative control mice in this study. dpi, days post infection; H&E, hematoxylin and eosin. Source data

In mice infected with omicron BA.1, those treated with P2-1B1, P5S-2B10 or P5-1H1 remained healthy, maintained body weight and survived infection, while 1 of 12 untreated control mice succumbed to disease on day 11 after challenge (Fig. 4a–c). P2-1B1 or P5S-2B10 treated mice had no detectable levels of live viruses in the lungs on day 3 post infection while untreated mice reached an average of 10^3^ PFU per lung (Fig. 4d,e). P5-1H1 protected against disease and body weight loss (Fig. 4c), but had moderate effect on reducing viral load in the lungs, consistent with its reduced neutralizing activity against BA.1 in vitro (Fig. 4f). No detectable levels of live viruses were found in the brain in P2-1B1, P5S-2B10 or P5-1H1 treated or untreated mice (Fig. 4d–f). These results indicated that P2-1B1 and P5S-2B10 conferred stronger protection than P5-1H1 against omicron BA.1 infection in vivo via prophylactic interventions.

In mice infected with beta, P2S-2E9 treated mice remained healthy and survived infection for 14 d, while the untreated group exhibited progressive body weight loss from 3 to 5 d post infection and all succumbed to infection by 5 d post infection (Fig. 4g). No detectable levels of live viruses were found in the lungs or brain of P2S-2E9 treated mice on day 4 post infection (Fig. 4h). By contrast, substantial levels of live viruses were detected in the lungs and brain in three of four untreated mice analyzed (Fig. 4h).

Immunohistochemistry analysis on day 3 (omicron BA.1) post infection showed that the lung tissue of P2-1B1, P5S-2B10 or P5-1H1 treated mice remained intact, while the lung sections of untreated mice presented moderate damage and inflammation, with marked infiltration of inflammatory cells (Fig. 4i–k). In the P2S-2E9 group, no damage or pulmonary inflammation was found in lung tissues, while untreated mice showed severe inflammatory infiltrates and edema (Fig. 4l). Infected cells in the lung sections of mice were detected using an N protein-specific antibody (Fig. 4i–l). These results indicated the broad and potent neutralizing activity of bnAbs in vitro translated into strong protection in vivo.

### Structural basis for bnAbs

**Fig. 5:**
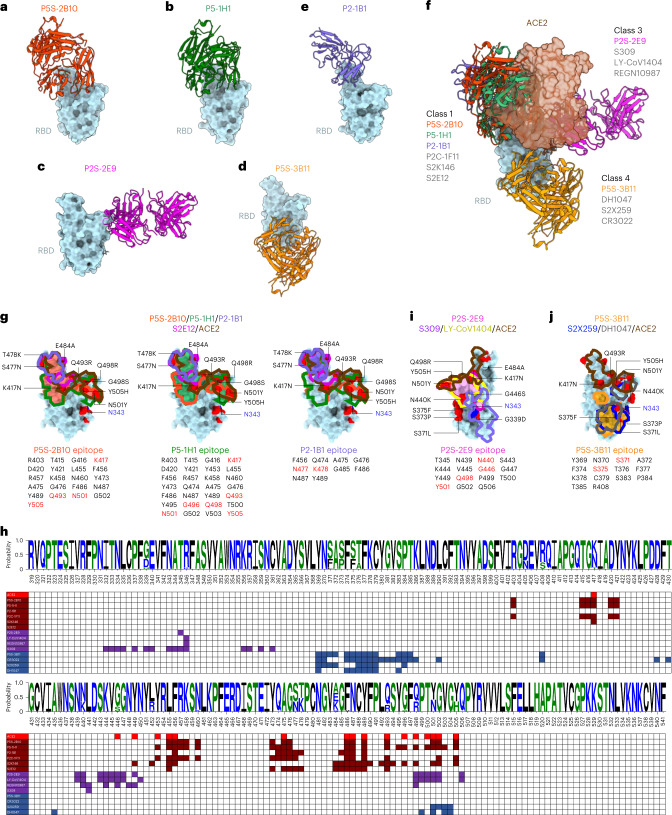
Binding mode and epitope specificity of five bnAbs to SARS-CoV-2. **a**–**e**, Crystal or cryo-EM structures of five Fab fragments complexed with RBDs derived from wild-type, beta or omicron BA.1. All RBDs are colored in cyan whereas P5S-2B10 (**a**) is in red, P5-1H1 (**b**) is in green, P2S-2E9 (**c**) is in magenta, P5S-3B11 (**d**) is in orange and P2-1B1 (**e**) is in purple. **f**, Fab fragments of P5S-2B10, P5-1H1, P2S-2E9, P5S-3B11 and P2-1B1 complexed with RBDs superimposed into one composite together with receptor ACE2 (brown). **g**, The footprints of P5S-2B10, P5-1H1 and P2-1B1 Fabs, together with those of S2E12 and ACE2, shown on the surface of the SARS-CoV-2 RBD. The epitope residues are indicted just below each structure, with the mutation sites found in omicron BA.1 indicated in red. **h**, Comparison of the epitope residues of the P5S-2B10, P5-1H1, P2-1B1, P2S-2E9 and P5S-3B11 antibodies with published representative antibodies and the receptor ACE2 along the linear RBD sequence. A logo plot of RBD sequences was created based on all tested SARS-CoV-2 variants. The numbering system follows that in the GISAID database. **i**,**j**, The footprints of P2S-2E9 (**i**) and P5S-3B11 (**j**) Fabs, together with those of representative antibodies and ACE2, shown on the surface of the SARS-CoV-2 RBD. The epitope residues are indicted just below each structure, with the mutation sites found in omicron BA.1 indicated in red. The highly conserved N-glycosylation residue N343 among the sarbecoviruses is indicated.

**Extended Data Fig. 5:**
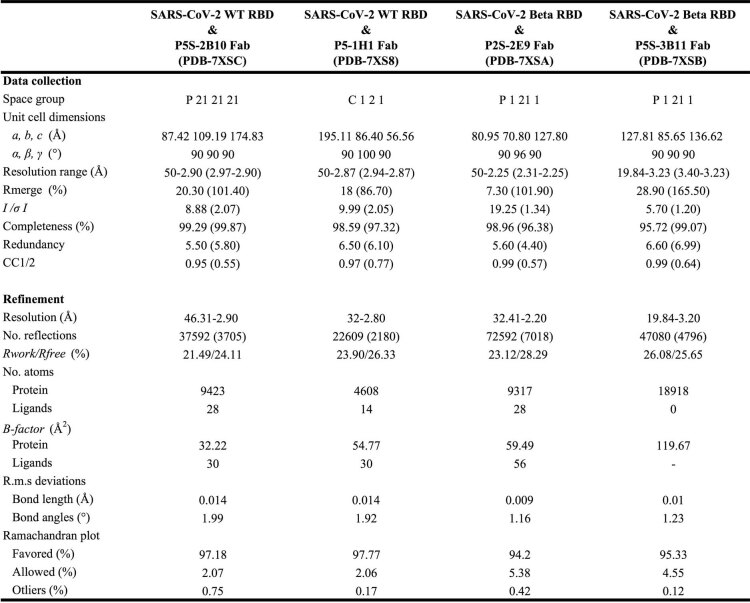
Data collection and refinement statistics (molecular replacement). This summary contained four crystal structures, SARS-CoV-2 wild-type (WT) RBD in complex with Fab P5S-2B10, Fab P5-1H1 and SARS-CoV-2 beta RBD in complex with Fab P2S-2E9, Fab P5S-3B11.

**Extended Data Fig. 6:**
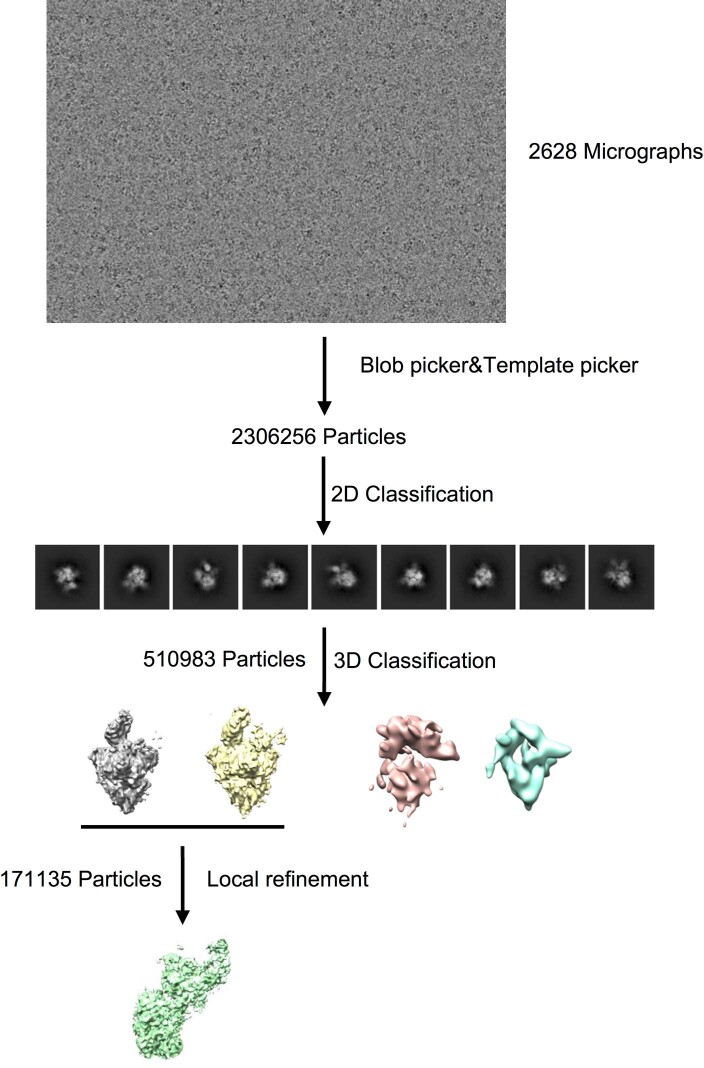
Flowchart for SARS-CoV-2 BA.1 S trimer in complex with Fab P2-1B1. The flowchart of cryo-EM data processing only exhibited key step, including representative electron micrograph, 2D class averages, 3D classification density map and local refinement density map.

**Extended Data Fig. 7:**
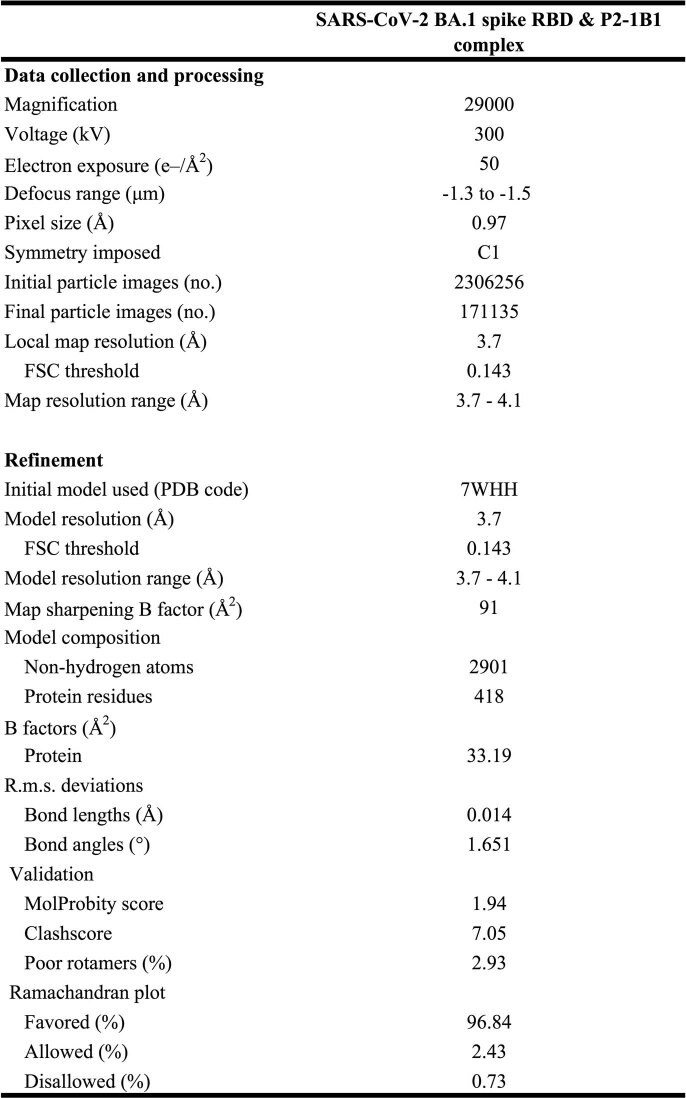
Cryo-EM data collection and processing, model building and refinement statistics. The statistics of model building and refinement based on the local refinement density map, the structure composing by BA.1 spike RBD and Fab P2-1B1.

We determined the crystal structures of four RBD–antibody complexes (P5S-2B10, P5-1H1, P2S-2E9 and P5S-3B11) and one cryo-electron microscopy (cryo-EM) structure of S–antibody complex (P2-1B1) (Extended Data Figs. 5–7). P5S-2B10 and P5-1H1 (group 1) bound to the wild-type RBD were determined at 2.88-Å and 2.79-Å resolution, and P2S-2E9 (group 1) and P5S-3B11 (group 2) bound to the beta RBD were determined at 2.20-Å and 3.20-Å resolution (Fig. 5a–d). P2-1B1 (group 1) bound to the omicron BA.1 S trimer was determined by cryo-EM with a resolution of 3.6 Å at the interface between P2-1B1 and RBD, allowing for model building and epitope mapping (Fig. 5e and Extended Data Figs. 6 and 7). Aligning and superimposing these five structures into one composite indicated that P5S-2B10, P5-1H1 and P2-1B1 exhibited a nearly identical binding pose to the top face of RBD, mimicking the binding mode of ACE2 (Fig. 5f). P2S-2E9 and P5S-3B11 targeted the outer face and inner face of RBD, respectively, distinct from the ACE2 binding site (Fig. 5f). P5S-2B10, P5-1H1 and P2-1B1 shared a similar binding mode with P2C-1F11, S2E12 and S2K146, three class 1 antibodies^20–22^ (Fig. 5f). P2S-2E9 bound to the region that overlapped with the epitopes of antibodies S309, LY-CoV1404 and REGN10987, which are typical class 3 or RBD-5 antibodies that bind to the solvent-exposed outer face of RBD^20,23–26^ (Fig. 5f). P5S-3B11, similar to DH1047, S2X259 and CR3022 (class 4 or RBD-7 antibodies), bound to the cryptic inner face of RBD^26–29^ (Fig. 5f).

**Extended Data Fig. 8:**
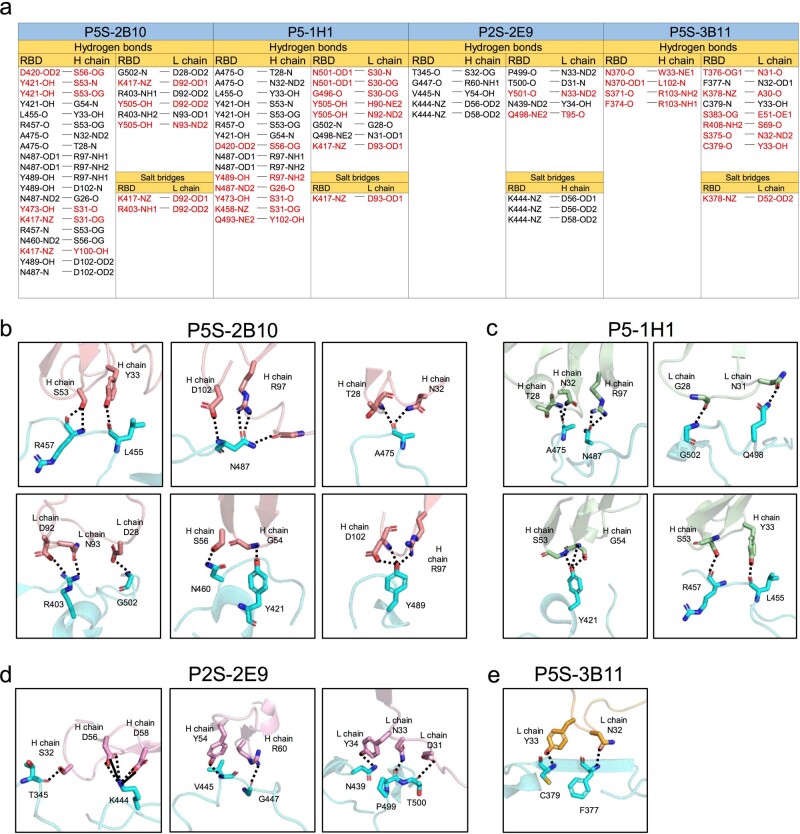
Hydrogen bonds and salt bridges formed between antibodies and SARS-CoV-2 RBD. **(a)** Those potentially affected by omicron BA.1 mutations are highlighted in red. **b-e**, Zoomed-in view of the hydrogen bonds and salt bridges as well as residues involved from P5S-2B10 **(b)**, P5-1H1 **(c)**, P2S-2E9 **(d)** and P5S-3B11 **(e)** antibodies and SARS-CoV-2 RBD are shown.

**Extended Data Fig. 9:**
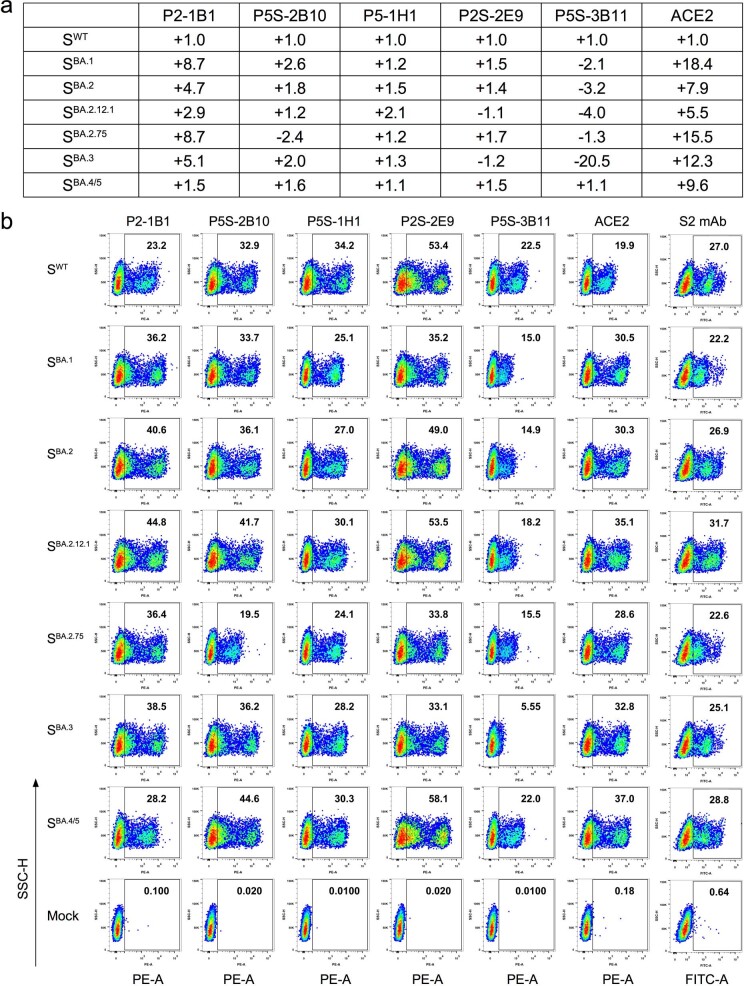
Antibody binding to cell-surface expressed spikes of wild-type, omicron BA.1, BA.2, BA.2.12.1, BA.2.75, BA.3, and BA.4/5. Binding of the five testing mAbs and ACE2 to the S protein expressed on the surface of HEK 293T, measured by flow cytometry. Fold changes in binding activity were measured by mean fluorescence intensity (MFI) relative to that of wild-type and summarized in **(a)**. The symbol ‘+’ and ‘−‘ indicate increase and decrease in binding, respectively. The result was calculated from two independent experiments. **(b)** The flow cytometry shown was representative of two independent experiments. Anti-S2 is a S2-specific antibody used for positive and normalized control. This mouse anti-S2 monoclonal antibody specifically targeted the epitope (1129-VIGIVNNTVYDPLQPELDSF-1148), which was identical among all tested SARS-CoV-2 variants. The numbers highlighted in the gates represent the percent of positive cells detected by indicated antibodies or ACE2.

We first analyzed the epitopes of P5S-2B10 and P5-1H1 revealed by X-ray crystallography with relatively high resolution compared with the epitope of P2-1B1 revealed by cryo-EM. P5S-2B10 and P5-1H1 had extensive overlaps in their epitopes (Fig. 5g,h). P5S-2B10 had a buried surface area of ~900 Å^2^ (21 residues), which was exclusively covered within the ~1,100-Å^2^ (28 residues) surface area buried by P5-1H1 (Fig. 5g). P5S-2B10 and P5-1H1 had extensive hydrophilic interactions with the wild-type RBD (Extended Data Fig. 8). At the binding interfaces, P5S-2B10 formed 26 hydrogen bonds and two salt bridges, whereas P5-1H1 had 23 hydrogen-bonding and one salt-bridge interaction with the RBD (Extended Data Fig. 8). The footprints of P5S-2B10 and P5-1H1 overlapped extensively with those of ACE2 and P2C-1F11, but remained rather different from other class 1 antibodies such as S2K146 and S2E12 (Fig. 5g,h). The omicron BA.1 had four substitutions (K417N, Q493R, N501Y and Y505H) in the P5S-2B10 epitope and six substitutions (K417N, Q493R, G496S, Q498R, N501Y and Y505H) in the P5-1H1 epitope (Fig. 5g). However, the overall binding capacity of P5S-2B10 or P5-1H1 to the surface-expressed omicron BA.1 S (S^BA.1^) protein was slightly higher or not affected by these substitutions, respectively, compared with that to the S^WT^ protein (Extended Data Fig. 9).

**Extended Data Fig. 10:**
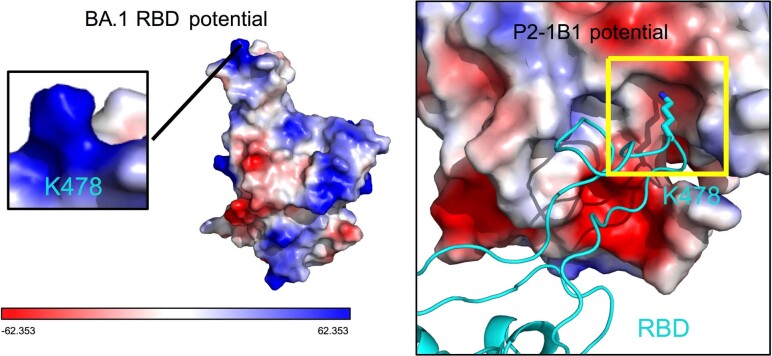
Electrostatic potential maps of the omicron BA.1 RBD (left) and P2-1B1 (right). The BA.1 RBD is shown by a cyan ribbon in the right panel and the side chain of mutation residue K478 is shown by stick.

Due to the relatively low resolution, the epitope of P2-1B1 could only be described in broad terms. The epitope buried a surface area of ~739 Å^2^ and contained ten residues (F456, Q474, A475, G476, N477, K478, G485, F486, N487 and Y489) (Fig. 5g). The P2-1B1 epitope contained two mutations (S477N and T478K) on the omicron BA.1 RBD, fewer than the four and six mutations in the epitopes of P5S-2B10 and P5-1H1 (Fig. 5g). In particular, the T478K substitution introduced a positively charged side chain and switched the electrostatic potential of this patch from neutral to positive (Extended Data Fig. 10). The P2-1B1 paratope surface interacting with the RBD K478 patch is negatively charged, and the electrostatic interaction is therefore expected to favor the binding of P2-1B1 to BA.1 RBD (Extended Data Fig. 10). As a result, the neutralizing activity of P2-1B1 against the BA.1 pseudovirus (12.1 ng ml^−1^) was slightly better than that of P5S-2B10 (18.5 ng ml^−1^), and was ~10-fold better than that of P5-1H1 (143.1 ng ml^−1^) (Fig. 2).

P2S-2E9 buried a surface area of ~770 Å^2^ and consisted of 15 residues on the solvent-exposed outer face of beta RBD (Fig. 5i). The omicron BA.1 had four substitutions (N440K, G446S, Q498R and N501Y) in the P2S-2E9 epitope (Fig. 5i), expected to affect at least 2 of the 13 hydrogen bonds at the binding interface (Extended Data Fig. 8). However, binding to the surface-expressed S^BA.1^ protein was not affected despite its reduced neutralization to BA.1 compared with that to wild-type D614G (Extended Data Fig. 9 and Fig. 2), suggesting the reduction in neutralization was likely a collective effect beyond binding alone. It is also possible that the enhanced binding of omicron variants to receptor ACE2 (5.5-fold to 18.4-fold) rendered their competitive advantage over P2S-2E9, and indirectly compromised the neutralizing activity of P2S-2E9 (Extended Data Fig. 9). The footprint of P2S-2E9 extensively overlapped with that of LY-CoV1404 but was rather distinctive from that of REGN10987 and S309 (Fig. 5h,i).

P5S-3B11 buried a surface area of ~810 Å^2^ and consisted of 14 residues on the cryptic inner face of beta RBD (Fig. 5j). Most of the omicron variants had two substitutions (S371L/F and S375F) in the P5S-3B11 epitope and were expected to affect 11 of the total 13 hydrogen bonds and salt bridges formed with RBD (Extended Data Fig. 8). As these mutations were located within the epitope, their impact on P5S-3B11 was more likely mediated through a direct rather than indirect fashion, similar to that found for P2S-2E9 (Extended Data Fig. 9 and Fig. 2). Many antibodies with similar epitopes to P5S-3B11, such as S2X259 and DH1047, were also compromised by substitutions found in omicron subvariants (Fig. 5h,j)^3,5^, largely due to the main-chain conformational change of the residing loop (Y369-C379) by triple S371L/S373P/S375F substitutions^9,30^. Overall, the structural basis for five representative bnAbs (P5S-2B10, P5-1H1, P2-1B1, P2S-2E9 and P5S-3B11) revealed the binding modes and potential mechanisms of action against SARS-CoV-2 variants.

## Discussion

In this report, we dissected the antibody responses of nine convalescent patients enrolled during the first wave of the COVID-19 pandemic in early 2020. We provided strong and solid evidence of the existence of a small number (7 of 476) of S^WT^-specific antibodies with broad neutralizing activities against all variants tested, including omicron subvariants BA.1, BA.2, BA.2.12.1, BA.2.75, BA.3 and BA.4/5, despite marked reduction or complete loss of neutralizing activity in the overwhelming majority of the remaining mAbs. Of the seven antibodies in group 1, five bound to the receptor-binding motif surface mimicking that of ACE2 and belonged to the class 1/RBS-A/RBD-2 community antibodies^15,16,20,26^. Four of these five antibodies favored the usage of the germline region IGHV3-53, while one used IGHV1-69. The preferential use of IGHV3-53 has been well documented among convalescent, messenger RNA-vaccinated and convalescent vaccinated individuals^16,18,19^. Such common features suggest that the IGHV3-53-encoded antibodies possess unique biochemical and structural features rendering them naturally strong in binding and highly complementary in shape to the receptor-binding motif surface. However, additional features are required to achieve broad and potent neutralizing activity, as IGHV3-53 usage alone does not guarantee their full activity. The other two antibodies in group 1 bound to the outer surface of RBD and belonged to the class 2/RBS-C/RBD-4 community (P5S-2A9) or class 3/RBS-D/RBD-5 community (P2S-2E9)^15,20,26^.

Crystal or cryo-EM structural analysis indicated these antibodies maintained their neutralizing breadth and potency either through tolerating substitutions within or close to their epitopes (P2-1B1, P5-1H1 and P2S-2E9) or through inadvertently increasing their binding potency to the mutated epitopes (P5S-2B10). It is possible that some of the mutations in omicron subvariants changed the local structure of the epitope, allowing better exposure and accessibility for the nAbs. Similar enhanced activities to omicron subvariants have also been reported for antibodies isolated or tested by other groups^5,10^. P2S-2E9 extensively overlapped in the footprints of and shared the same germlines, IGHV2-5 and IGLV2-14, with LY-CoV1404, the parental antibody of Bebtelovimab by Eli Lilly, which was also isolated from an early convalescent patient and demonstrates exceptional neutralizing breadth and potency against all VOCs identified, including omicron subvariants^5,9–11,24^. A single intraperitoneal injection of representative antibodies P2-1B1, P5S-2B10 and P5-1H1 in class 1 and P2S-2E9 in class 3 as a prophylactic treatment protected mice from infection with the most antigenically divergent variants, omicron BA.1 and beta. These results indicated that natural infection with wild-type SARS-CoV-2 could generate broadly neutralizing and protective mAbs targeting to multiple regions of the RBD, including omicron subvariants.

It is expected that more antibodies similar to the ones identified here and those targeting other regions of the RBD will be identified as more studies continue to dig deeper into the antibody repertories in convalescent and vaccinated individuals. Among these bnAbs are the IGHV1-58 supersite antibodies and those targeting the most conserved cryptic inner face of the RBD of SARS-CoV-2 variants, as well as other sarbecoviruses (class 4/RBD-6/RBD-7)^9,18,21,31^. However, the latter antibodies would have to overcome substitutions such as S371L/F, S373P, S375F and T376A found in the omicron subvariants in this region to achieve broad and potent neutralizing activity^3,5,8,9^. Nevertheless, identification of broadly neutralizing and protective antibodies in naturally infected individuals highlights the broad and diverse nAb repertoire established and imprinted by the wild-type SARS-CoV-2 infection^3–5,8,32^. Particularly, it also points to the RBD as a viable and critical target to induce a broad and protective response against VOCs, as recently demonstrated by several RBD-based vaccine strategies either in the form of a trimer or nanoparticles^33–35^. While studies on booster immunity are ongoing, it is reasonable to speculate that the broad and potent antibodies identified here and elsewhere in convalescent patients likely contribute to the hybrid immunity augmented by the vaccine booster among previously infected and recovered patients^36–40^. While these antibodies could serve as promising candidates for the development of next-generation antibody therapies, a deeper understanding of how the memory B cells producing these antibodies are initially induced, maintained and recalled will hold the key to rational design of vaccines capable of selectively boosting such desirable antibodies against SARS-CoV-2 variants and beyond.

While selectively boosting and recalling antibody response is practically challenging, several strategies are being proposed and evaluated in the field. Among these, a germline targeting strategy is being tested for HIV-1 vaccines (426c and eOD-GT8) to selectively target the antibody germline IGHV1-2 to induce VRC01-like antibodies, which showed broadly neutralizing activities against a diverse panel of HIV-1 variants around the world^41–44^. Analogously, designing SARS-CoV-2 antigens to target the germline IGHV3-53 or IGHV2-5 would be expected to stimulate the type of rare but broadly neutralizing antibodies found in the current study. Glycan engineering is another strategy being tested to reduce the immunogenicity of regions outside the conserved epitopes. This could be achieved through the addition of N-linked glycan and/or polyethylene glycol (PEG). Particularly, as the N-linked glycosylation motif (Asn-X-Ser/Thr) can be introduced into the spike or RBD of SARS-CoV-2, glycan silencing could be precisely designed to shield unwanted epitopes, while improving the exposure of desirable epitopes for broadly neutralizing antibodies^45–49^. Finally, as our understanding of the structure of antigens and immunity to antigens improves, it will provide additional concepts and technologies to selectively target and amplify the type of broadly neutralizing antibodies identified here.

## Methods

### Study approval, convalescent patients and blood samples

The study was approved by the Research Ethics Committee of Shenzhen Third People’s Hospital, China (approval no.: 2020-084). The research was conducted in strict accordance with the rules and regulations of the Chinse government for the protection of human subjects. All participants had provided written, informed consents for sample collection and subsequent analysis. Detailed information of participants in this study is provided in Supplementary Table 1. The study enrolled a total of nine patients aged 32–73 yr and recovered from infection with wild-type SARS-CoV-2 in January 2020. Of these, three (P2, P5 and P10) once developed severe pneumonia whereas the remaining six (P43, P75, P104, P140, P186 and P195) only had mild symptoms during hospitalization at Shenzhen Third People’s Hospital. P2 and P5 donated their blood samples twice and the remaining patient once during a 16–111-d recovery period post symptom onset. According to the policies at that time, local patients with COVID-19 were given free treatments and follow-up visits. The collected blood samples were separated into plasma and PBMCs through Ficoll-Hypaque density gradient centrifugation. The plasma samples were heat-inactivated at 56 °C for 1 h and stored at −80 °C, whereas the PBMCs were maintained in freezing media and stored in liquid nitrogen until use.

### Production of pseudoviruses and neutralizing assay

The wild-type pseudovirus used throughout the analysis was the wild-type strain (GenBank: MN908947.3) or had a D614G mutation (D614G). The alpha variant (Pango lineage B.1.1.7, GISAID: EPI_ISL_601443) included a total of nine reported mutations in the S protein (del69-70, del144, N501Y, A570D, D614G, P681H, T716I, S982A and D1118H). The beta variant (Pango lineage B.1.351, GISAID: EPI_ISL_700450) included ten identified mutations in the spike (L18F, D80A, D215G, del242-244, S305T, K417N, E484K, N501Y, D614G and A701V). The gamma variant (Pango lineage P.1, GISAID: EPI_ISL_792681) had 12 reported mutations in the spike (L18F, T20N, P26S, D138Y, R190S, K417T, E484K, N501Y, D614G, H655Y, T1027I and V1176F). The delta variant (Pango lineage B.1.617.2, GISAID: EPI_ISL_1534938) included ten reported mutations in the spike (T19R, G142D, del156-157, R158G, A222V, L452R, T478K, D614G, P681R and D950N). The delta plus variant (Pango lineage AY.x, GISAID: EPI_ISL_ 3019629) had one more mutation, K417N, than the delta variant. The epsilon variant (Pango lineage B.1.429, GISAID: EPI_ISL_2922315) included S13I, W152C, L452R and D614G in the spike. The kappa variant (Pango lineage B.1.617.1, GISAID: EPI_ISL_1384866) included T95I, G142D, E154L, L452R, E484Q, D614G, P681R and N1071H in the spike. The mu variant (Pango lineage B.1.621, GISAID: EPI_ISL_3987640) included T95I, Y144T, Y145S, ins146N, R346K, E484K, N501Y, D614G, P681H and D950N in the spike. The eta variant (Pango lineage B.1.525, GISAID: EPI_ISL_2885901) included Q52R, A67V, del69-70, del144, E484K, D614G, Q677H and F888L in the spike. The iota variant (Pango lineage B.1.526, GISAID: EPI_ISL_2922249) included L5F, T95I, D253G, E484K, D614G and A701V in the spike. The omicron BA.1 variant (Pango lineage BA.1, GISAID: EPI_ISL_6752027) was constructed with 34 mutations in the spike (A67V, del69-70, T95I, G142D, del143-145, del211, L212I, ins214EPE, G339D, S371L, S373P, S375F, K417N, N440K, G446S, S477N, T478K, E484A, Q493R, G496S, Q498R, N501Y, Y505H, T547K, D614G, H655Y, N679K, P681H, N764K, D796Y, N856K, Q954H, N969K and L981F). The omicron BA.2 variant (Pango lineage BA.2, GISAID: EPI_ISL_8515362) was constructed with 29 mutations in the spike (T19I, del24-26, A27S, G142D, V213G, G339D, S371F, S373P, S375F, T376A, D405N, R408S, K417N, N440K, S477N, T478K, E484A, Q493R, Q498R, N501Y, Y505H, D614G, H655Y, N679K, P681H, N764K, D796Y, N969K and Q954H). BA.2.12.1 spike was constructed based on BA.2 with additional L452Q and S704. BA.2.75 spike was constructed based on BA.2 with additional W152R, F157L, I210V, G257S, D339H, G446S, N460K and Q493R (reversion). The omicron BA.3 variant (Pango lineage BA.3, GISAID: EPI_ISL_7740765) was constructed with 30 mutations in the spike (A67V, del69-70, T95I, G142D, del143-145, del211, L212I, G339D, S371F, S373P, S375F, D405N, K417N, N440K, G446S, S477N, T478K, E484A, Q493R, Q498R, N501Y, Y505H, D614G, H655Y, N679K, P681H, N764K, D796Y, Q954H and N969K). The omicron BA.4 variant (Pango lineage BA.4, GISAID: EPI_ISL_12559461) was constructed with 32 mutations in the spike (T19I, del24-26, A27S, del69-70, G142D, V213G, G339D, S371F, S373P, S375F, T376A, D405N, R408S, K417N, N440K, G446S, L452R, S477N, T478K, E484A, F486V, Q498R, N501Y, Y505H, D614G, H655Y, N679K, P681H, N764K, D796Y, Q954H and N969K). BA.4 and BA.5 shared the same amino acid sequence in the spike. The full-length genes of spike variants were synthesized by Genwiz and verified by sequencing.

Pseudoviruses were generated by cotransfecting HEK 293T cells (ATCC) with human immunodeficiency virus backbones expressing firefly luciferase (pNL4-3-R-E-luciferase) and pcDNA3.1 vector encoding either wild-type or variant S proteins^51,52^. Viral supernatant was collected 48 h or 72 h later, centrifuged to remove cell lysis and stored at −80 °C until use. Viral infectious titers were measured by luciferase activity in the HeLa-hACE2 cells using Bright-Glo Luciferase Assay Vector System (Promega). Berthold Centro LB 960 was used for measuring luciferase activity. Neutralization assays were performed by incubating pseudoviruses with serial dilutions of heat-inactivated plasma or purified mAbs at 37 °C for 1 h. Approximately 1.5 × 10^4^ per well of HeLa-hACE2 cells were then added in duplicate to the above virus–antibody mixture. At 48 h later, the half-maximal inhibitory dilution of plasma (ID_50_) or concentration of the mAbs (IC_50_) was determined by luciferase activity using GraphPad Prism 8.3 (GraphPad Software). HeLa-hACE2 cells were kindly provided by Q. Ding at the Center for Infectious Research of Tsinghua University.

### Plasma and antibody binding analyzed by ELISA

The recombinant S, S1, RBD and S2 proteins derived from the wild-type SARS-CoV-2, and S1 proteins or S proteins of SARS-CoV-1 and MERS-CoV (Sino Biological), were diluted to final concentrations of 0.5 or 2 μg ml^−1^, coated onto 96-well plates and incubated overnight at 4 °C. The plates were washed with PBS-T (PBS containing 0.05% Tween 20) and blocked with blocking buffer (PBS containing 5% skim milk and 2% BSA) at room temperature for 1 h. Serially diluted plasma samples or mAbs were added to the plates and incubated at 37 °C for 1 h. After extensive washing, the plates were then incubated with secondary anti-human IgG labeled with HRP (1:5,000 dilution) (ZSGB-BIO) at 37 °C for 30 min or 1 h before incubation with TMB substrate (Kinghawk) at room temperature for 5 min or 20 min. Optical density was measured by a spectrophotometer at 450 nm.

### Isolation of S^WT^-specific single B cells by FACS

SARS-CoV-2 S^WT^-specific B cells were sorted as previously described^53,54^. In brief, PBMCs from convalescent individuals were collected and incubated with an antibody and recombinant S^WT^ trimer cocktail for identification of S^WT^-specific B cells. The cocktail consisted of CD19-PE-Cy7 (1:50 dilution), CD3-Pacific Blue (1:50 dilution), CD8-Pacific Blue (1:25 dilution), CD14-Pacific Blue (1:50 dilution), CD27-APC-H7 (1:25 dilution), IgG-FITC (1:12.5 dilution) (or IgM−PerCP-Cy5.5 (1:50 dilution), IgD−PE-CF594 (1:25 dilution)) (BD Biosciences) and recombinant S^WT^-Strep or S^WT^-His purified in our laboratory. Three consecutive staining steps were conducted. The first was using a LIVE/DEAD Fixable Dead Cell Stain Kit (Invitrogen) to exclude the dead cells. The second was mixing with an antibody and recombinant S^WT^ trimer cocktail to identify S^WT^-specific B cells. The third was to target the recombinant S^WT^ trimer captured on the surface of B cells by either Streptavidin-APC (eBioscience) or anti-his-APC/PE antibodies (1:25 dilution) (Abcam). The stained cells were thoroughly washed and resuspended in PBS before being strained through a 70-μm cell mesh (BD Biosciences). SARS-CoV-2 S^WT^-specific single B cells were gated as either CD19^+^CD3^−^CD8^−^CD14^−^CD27^+^IgG^+^S^WT+^ or CD19^+^CD3^−^CD8^−^CD14^−^IgM^−^IgD^−^S^WT+^ and sorted (BD Aria II) into the 96-well PCR plates containing 20 μl of lysis buffer (5 μl of 5 × first strand buffer, 0.5 μl of RNaseOUT, 1.25 μl of 0.1 M dithiothreitol (Invitrogen) and 0.0625 μl of Igepal (Sigma) per well). Plates were then snap-frozen on dry ice and stored at −80 °C until the reverse transcription reaction.

### Single B cell PCR, cloning and expression of mAbs

The IgG heavy and light chain variable genes were amplified by nested PCR and cloned into linear expression cassettes to produce full IgG1 antibodies as previously described^54,55^. Specifically, all second round PCR primers containing tag sequences were used to produce the linear immunoglobulin expression cassettes by overlapping PCR. Meanwhile, the variable genes of heavy and light chain were sequenced, synthesized and then cloned into the backbone of antibody expression vectors containing the constant regions of human IgG1 by GenScript^56^. Overlapping PCR products of paired heavy and light chain expression cassettes were cotransfected into the HEK 293T cells (ATCC) to produce antibodies for binding analysis. Large quantities of mAbs were produced by transient transfection of 293F cells (Life Technologies) with equal amounts of paired heavy and light chain plasmids. Antibodies in the culture supernatant were purified by affinity chromatography using Protein A bead columns (GE Healthcare) and their concentrations were determined by a NanoDrop2000 (Thermo Scientific).

### Gene family usage and recombination analysis of mAbs

The IMGT/V-QUEST program (http://www.imgt.org/IMGT_vquest/vquest) was used to analyze the germline gene, degree of somatic hypermutation, framework region and the loop lengths of complementarity determining region 3 (CDR3) of each antibody. Chord diagrams showing the germline gene usages of paired heavy and light chain were analyzed and presented by the R package circlize v.0.4.14. The width of the linking arc is proportional to the number of antibodies identified.

### Epitope mapping by competition surface plasmon resonance

For epitope mapping, two different mAbs were sequentially injected and monitored for binding activity to determine whether the two mAbs recognized separate or closely situated epitopes. To determine competition with the human ACE2, antibodies (1 μM) were injected onto the RBD-immobilized CM5 chip until the binding steady-state was reached. ACE2 (2 μM) was then injected for 60 s. Blocking efficacy was determined by comparison of response units with and without previous antibody incubation. Biacore 8K Control Software v.3.0.12.15655 was used for binding competition studies.

### Crystallization and data collection

The SARS-CoV-2 RBD and the Fab fragment were mixed at a molar ratio of 1:1.2, incubated at 4 °C for 2 h and further purified by gel-filtration chromatography. The purified complex concentrated to approximately 10–15 mg ml^−1^ in HBS buffer (10 mM HEPES, pH 7.2, 150 mM NaCl) was used for crystallization. The screening trials were performed at 18 °C using the sitting-drop vapor-diffusion method by mixing 0.2 μl of protein with 0.2 μl of reservoir solution. Crystals of P5S-2B10 Fab and RBD complex were successfully obtained in 0.2 M magnesium sulfate heptahydrate, 17% w/v PEG 3350, whereas P5-1H1 Fab and RBD complex was obtained in 2% v/v Tacsimate pH 5.0, 0.1 M sodium citrate tribasic dihydrate pH 5.4 and 13% w/v PEG 3350. P2S-2E9 Fab and RBD complex was obtained in 15% v/v 2-Propanol, 0.1 M sodium citrate tribasic dihydrate pH 4.8, 11% w/v PEG 10000, and P5S-3B11 Fab and RBD complex in 0.05 M citric acid pH 4.4, 0.05 M BIS-TRIS propane, 16% w/v PEG 3350. All crystals were collected, soaked briefly in mother liquid with 20% glycerol and flash-frozen in liquid nitrogen. Diffraction data were collected at a wavelength of 0.987 Å on the BL18U1 beam line of the Shanghai Synchrotron Research Facility and processed by HKL2000. The data processing statistics are listed in Extended Data Fig. 5.

### Structure determination and refinement

The structure was determined by the molecular replacement method with PHASER in CCP4 suite 7.1.007 (ref. ^57^). The search models were the SARS-CoV-2 RBD structure (PDB: 6M0J) and the structures of the variable domains of the heavy and light chains available in the PDB with the highest sequence identities. Subsequent model building and refinement were performed using COOT v.0.9.2 and PHENIX v.1.18.2, respectively^58,59^. The structural refinement statistics are listed in Extended Data Fig. 5. All structural figures were generated using PyMOL 2.0 and Chimera v.1.15.

### Cryo-EM structural determination

Aliquots of complexes (4 μl, in buffer containing 20 mM Tris, pH 8.0, and 150 mM NaCl) of SARS-CoV-2 BA.1 S ectodomains (2 mg ml^−1^) and P2-1B1 Fab were applied to glow-discharged holey carbon grids (Quantifoil grid, Cu 300 mesh, R1.2/1.3). Fab fragments were mixed with SARS-CoV-2 S trimer at a molar ratio of 1.2:1. The grids were then blotted for 3 s and immediately plunged into liquid ethane using Vitrobot Mark IV (Thermo Fisher Scientific). The cryo-EM data of complexes were collected by the FEI Titan Krios microscope (Thermo Fisher Scientific) at 300 kV with a Gatan K3 Summit direct electron detector (Gatan) at Tsinghua University. In total, 2,628 movies were collected by SerialEM version 4.0.4, with a magnification of 29,000 and defocus range between −1.3 and −1.5 μm. Each movie has a total accumulated exposure of 50 e^−^ Å^−2^ fractionated in 32 frames of 2.13-s exposure. The stacks were binned twofold, resulting in a pixel size of 0.97 Å per pixel. Motion correction (MotionCor2 v.1.2.6), CTF estimation (GCTF v.1.18) and nontemplated particle picking (Gautomatch v.0.56; http://www.mrc-lmb.cam.ac.uk/kzhang/) were automatically executed using the TsingTitan.py program^60,61^. Sequential data processing was carried out with cryoSPARC v.3.3.1 (refs. ^62,63^). The initial models of SARS-CoV-2 BA.1 RBD (PDB: 7WHH) and P2-1B1 Fab were fitted to the map using UCSF Chimera v.1.15 (ref. ^64^). Manual model rebuilding was carried out with COOT v.0.9.2 and refined with PHENIX v.1.18.2 real-space refinement. The quality of the final model was analyzed by PHENIX v.1.18.2. The validation statistics of the structural models are summarized in Extended Data Fig. 7. All structural figures were generated using PyMOL 2.0 and Chimera v.1.15.

### Binding of mAbs to cell-surface-expressed S proteins

The entire procedure was conducted as previously published^51,52,65^. Specifically, HEK 293T cells were transfected with expression plasmids encoding either wild-type or omicron variant S proteins, and incubated at 37 °C for 36 h. Cells were digested from the plate with trypsin and distributed onto 96-well plates. Cells were washed twice with 200 µl of staining buffer (PBS with 2% heated-inactivated FBS) between each of the following steps. First, cells were stained with each antibody (1 μg ml^−1^), or ACE2 (1 μg ml^−1^), or S2-specific mAb (1 μg ml^−1^) (1:200 dilution) (MP Biomedicals) at 4 °C for 30 min. PE-labeled anti-human IgG Fc (1:40 dilution; BioLegend), anti-his PE secondary antibody (1:200 dilution; Miltenyi) or anti-mouse IgG FITC (1:200 dilution; Thermo Fisher Scientific) was added and incubated at 4 °C for 30 min. After extensive washes, the cells were resuspended and analyzed with BD LSRFortessa (BD Biosciences) and FlowJo 10 software (FlowJo). HEK 293T cells with mock transfection were stained as background control. Fold changes in antibody binding were calculated by the ratio of the total fluorescence intensity (TFI) of omicron over wild-type, normalized by that of S2-specific antibody (nTFI). TFI was calculated by multiplying the mean fluorescence intensity (MFI) and the number of positive cells in the selected gates. For example, the fold change in BA.1 of P5S-2B10 was calculated by the following formula: fold change = (BA.1 of TFI/BA.1 of nTFI)/(wild-type of TFI/wild-type of nTFI). TFI = MFI × subset frequency.

### Antibody protection in hACE2 transgenic mice

Mouse experiments were performed in a Biosafety Level 3 (BSL-3) facility in accordance with the National University of Singapore (NUS) Institutional Animal Care and Use Committee (protocol no. R20-0504), and the NUS Institutional Biosafety Committee and NUS Medicine BSL-3 Biosafety Committee approved SOPs. As previously described^66^, 8-week-old female K18-hACE2 transgenic mice (InVivos) were utilized for this study. The mice were housed and acclimatized in a BSL-3 facility for 72 h before the start of the experiment. The housing conditions were 23 ± 2 °C (high/low temperature), 50 ± 10% (high/low humidity) and 12-h light/12-h dark (light cycle). K18-hACE2 transgenic mice were subjected to P2-1B1, P5S-2B10, P5-1H1 or P2S-2E9 (10 mg kg^−1^) delivered through intraperitoneal injection a day before infection or left untreated. P5S-2B10 and P5-1H1 experiments shared a group of untreated mice. The viral challenge was conducted through intranasal delivery in 25 μl of 1.7 × 10^3^ PFU of the infectious SARS-CoV-2 omicron BA.1 or beta variant. Body weights were measured before infection as baseline and monitored daily throughout the following 14 d. Mice were euthanized when their body weight fell below 80% of their baseline body weight. Some of the mice from each experimental group were killed at 3 d for omicron BA.1 or 4 d for the beta variant post infection, and lung and brain tissues were collected. Each organ was halved for the plaque assay and histology analysis, respectively.

For virus titer determination, supernatants from homogenized tissues were tenfold serially diluted in DMEM supplemented with antibiotic and antimycotic, and added to A549-hACE2 cells (omicron virus) or Vero E6 cells (beta virus) in 12-well plates. The inoculum was removed after 1 h of incubation for virus adsorption. Cells were washed once with PBS before 1.2% MCC-DMEM overlay medium was added to each well. Then cells were incubated at 37 °C, 5% CO_2_ for 72 h for plaque formation. Cells were fixed in 10% formalin overnight and counterstained with crystal violet. The number of plaques was determined and the virus titers of individual samples were expressed as logarithm of PFU per organ.

For histopathological analyses, lung lobes were fixed in 3.7% formaldehyde solution before removal from BSL-3 containment. Tissues were routinely processed, embedded in paraffin blocks (Leica Surgipath Paraplast), sectioned at 4-μm thickness and stained with hematoxylin and eosin (Thermo Scientific) following standard histological procedures. For immunohistochemistry, the sections were deparaffinized and rehydrated, followed by heat-mediated antigen retrieval, quenching of endogenous peroxidases and protein blocking. Sections were then covered with rabbit anti-SARS-CoV-2 N protein mAb (Abcam, 1:1,000 dilution) for 1 h at room temperature. Subsequently, sections were incubated with rabbit-specific HRP polymer secondary antibody (Abcam, no dilution), visualized using chromogenic substrate DAB solution (Abcam) and counterstained with hematoxylin.

### Data reporting

No statistical methods were used to predetermine sample sizes but our sample sizes are similar to those reported in previous publications. The sample size of nine COVID-19 convalescent patients is sufficient for isolating nAbs in the field (PMID: 32454513 and PMID: 32698192). For animal experiments, the number of mice for the in vivo protection assay in each group was 4–6, which is acceptable in the field (PMID: 33657424 and PMID: 33431856). Data distribution was assumed to be normal but this was not formally tested. The experiments for antibody isolation from COVID-19 convalescents were not randomized. Mice in antibody protection experiments were randomly divided into treated and untreated groups. Data collection and analysis were not performed blind to the conditions of the experiments. No animals or data points were excluded.

### Reporting summary

Further information on research design is available in the Nature Portfolio Reporting Summary linked to this article.

## Online content

Any methods, additional references, Nature Portfolio reporting summaries, source data, extended data, supplementary information, acknowledgements, peer review information; details of author contributions and competing interests; and statements of data and code availability are available at 10.1038/s41590-023-01449-6.

## Supplementary information

Reporting Summary

Supplementary Table Supplementary Table 1: Clinical characterization of the study subjects. Supplementary Table 2: Sequences of 40 anti-RBD monoclonal nAbs.

## Data Availability

Structure coordinates have been deposited in the Protein Data Bank under accession codes 7XSC (P5S-2B10:WT-RBD), 7XS8 (P5-1H1:WT-RBD), 7XSA (P2S-2E9:Beta-RBD) and 7XSB (P5S-3B11:Beta-RBD). Sequences of 40 RBD-specific mAbs have been provided in Supplementary Table 2. All data generated or analyzed during this study are available within the paper and the Supplementary Information files. Source data are provided with this paper.

## Source data

Source Data Statistical source data for Figs. 1, 3 and 4 and Extended Data Figs. 1, 2 and 3.
