# Supplementary material for: Infection with wild-type SARS-CoV-2 elicits broadly neutralizing and protective antibodies against omicron subvariants
Source: Nat Immunol. 2023 Mar 13;24(4):690–9. doi: 10.1038/s41590-023-01449-6 (PMC10063446; doi:10.1038/s41590-023-01449-6)
Supplement: Supplementary file 1 — Reporting Summary [file 41590_2023_1449_MOESM1_ESM.pdf]

Corresponding author(s): Linqi Zhang

Last updated by author(s): Jan 31, 2023

Reporting Summary

Nature Portfolio wishes to improve the reproducibility of the work that we publish. This form provides structure for consistency and transparency in reporting. For further information on Nature Portfolio policies, see our Editorial Policies and the Editorial Policy Checklist.

Statistics

For all statistical analyses, confirm that the following items are present in the figure legend, table legend, main text, or Methods section.

n/a	Confirmed	
☐	☒	The exact sample size (*n*) for each experimental group/condition, given as a discrete number and unit of measurement
☐	☒	A statement on whether measurements were taken from distinct samples or whether the same sample was measured repeatedly
☐	☒	The statistical test(s) used AND whether they are one- or two-sided<br>*Only common tests should be described solely by name; describe more complex techniques in the Methods section.*
☒	☐	A description of all covariates tested
☒	☐	A description of any assumptions or corrections, such as tests of normality and adjustment for multiple comparisons
☐	☒	A full description of the statistical parameters including central tendency (e.g. means) or other basic estimates (e.g. regression coefficient) AND variation (e.g. standard deviation) or associated estimates of uncertainty (e.g. confidence intervals)
☒	☐	For null hypothesis testing, the test statistic (e.g. *F*, *t*, *r*) with confidence intervals, effect sizes, degrees of freedom and *P* value noted<br>*Give P values as exact values whenever suitable.*
☒	☐	For Bayesian analysis, information on the choice of priors and Markov chain Monte Carlo settings
☒	☐	For hierarchical and complex designs, identification of the appropriate level for tests and full reporting of outcomes
☒	☐	Estimates of effect sizes (e.g. Cohen's *d*, Pearson's *r*), indicating how they were calculated

Our web collection on statistics for biologists contains articles on many of the points above.

Software and code

Policy information about availability of computer code

Data collection	Biacore 8K Control Software v3.0.12.15655 was used for binding competition studies. Berthold Centro LB 960 was used for measuring luciferase activity. Diffraction data were collected at a wavelength of 0.987 Å on the BL18U1 beam line of the Shanghai Synchrotron Research Facility (SSRF). HKL2000 was used for crystal data processing. PHASER version (CCP4 Interface 7.1.007) was used for molecular replacement to solve the complex structure. COOT v.0.9.2 was used for model building and refinement manually. Phenix v.1.18.2 was used for automatical model refinement. The Cryo-EM data of complex were collected by the FEI Titan Krios microscope (Thermo Fisher Scientific) at 300 kV with a Gatan K3 Summit direct electron detector (Gatan Inc.) at Tsinghua University. Flow cytometry BD Aria II and LSRFortassa were used for cell sorting and fluorescence analysis.
Data analysis	The program IMGT/V-QUEST (http://www.imgt.org/IMGT_vquest/vquest) was applied to analyze gene germline, complementarity determining region (CDR) 3 length, and somatic hypermutation (SHM). The CDR3 length was calculated from amino acids sequences. The SHM frequency was calculated from the mutated nucleotides. Graphs were presented by GraphPad Prism version 8.3, R package circlize v0.4.14, BIAcore 8K Evaluation v3.0.12.15655, PyMOL 2.0 and Chimera v.1.15 softwares. Flow cytometry data analysis was performed using FlowJo version 10 software. Motion correction (MotionCor2 v.1.2.6), CTF estimation (GCTF v.1.18), and non-templated particle picking (Gautomatch v.0.56; http://www.mrc-lmb.cam.ac.uk/kzhang/) were automatically executed using the TsingTitan.py program. Sequential data processing was carried out on cryoSPARC v3.3.1.

For manuscripts utilizing custom algorithms or software that are central to the research but not yet described in published literature, software must be made available to editors and reviewers. We strongly encourage code deposition in a community repository (e.g. GitHub). See the Nature Portfolio guidelines for submitting code & software for further information.

Data

Policy information about availability of data

All manuscripts must include a data availability statement. This statement should provide the following information, where applicable:

- Accession codes, unique identifiers, or web links for publicly available datasets
- A description of any restrictions on data availability
- For clinical datasets or third party data, please ensure that the statement adheres to our policy

Structure coordinate has been deposited in the Protein Data Bank under accession code 7XSC (P5S-2B10:WT-RBD), 7XS8 (P5-1H1:WT-RBD), 7XSA (P2S-2E9:Beta-RBD), and 7XSB (P5S-3B11:Beta-RBD). Sequences of 40 RBD-specific mAbs have been provided in the Supplementary Table 2. All data generated or analyzed during this study are available within the paper and the supplementary information files. Source data are provided with this paper.

Human research participants

Policy information about studies involving human research participants and Sex and Gender in Research.

Reporting on sex and gender	Sex and gender were not considered in study design.
Population characteristics	The study enrolled a total of nine patients aged between 32 to 73 years old and recovered from infection with wild-type SARS-CoV-2 in January 2020. Of which, three (P#2, P#5, and P#10) once developed severe pneumonia while the remaining six (P#43, P#75, P#104, P#140, P#186, and P#195) only had mild symptom during hospitalization at Shenzhen Third People's Hospital. P#2 and P#5 donated their blood samples twice while the remaining once during 16 to 111 days recovery period post the symptom onset. More detailed information was shown in Supplementary Table 1.
Recruitment	The study enrolled a total of nine patients aged between 32 to 73 years old and recovered from infection with wild-type SARS-CoV-2 in January 2020. These local COVID-19 patients were given free treatments and follow-up visits at Shenzhen Third People's Hospital. There were not any potential self-selection bias or other biases.
Ethics oversight	The study was approved by the Research Ethics Committee of Shenzhen Third People's Hospital, China (approval number: 2020-084).

Note that full information on the approval of the study protocol must also be provided in the manuscript.

Field-specific reporting

Please select the one below that is the best fit for your research. If you are not sure, read the appropriate sections before making your selection.

☒ Life sciences ☐ Behavioural & social sciences ☐ Ecological, evolutionary & environmental sciences

For a reference copy of the document with all sections, see nature.com/documents/nr-reporting-summary-flat.pdf

Life sciences study design

All studies must disclose on these points even when the disclosure is negative.

Sample size	We isolated antibodies from PBMCs of nine SARS-CoV-2-infected donors, including 3 severe patients and 6 mild patients, aging from 32 to 73 years old. No statistical methods were used to pre-determine the sample size. This sample size is sufficient for isolating neutralizing antibodies in the field (PMID: 32454513 and PMID: 32698192). For animal experiments, no sample-size calculation was performed. The number of mice in vivo protection assay in each group was 4 to 6, which is acceptable in the field (PMID: 33657424 and PMID: 33431856).
Data exclusions	No data were excluded.
Replication	ELISA were performed two times independently. Neutralization assay were performed at least two times independently. The cell staining assay for binding between mutated spike and antibody was performed two times independently. The epitope mapping experiments using competition SPR were performed two times independently. All attempts at replication were successful. Single cell sorting, structure elucidation, and animal experiments were performed once because of their own characteristics.
Randomization	There is no allocation in this study, so randomization is not applicable.
Blinding	No blinding was conducted since there was no specific grouping.

Reporting for specific materials, systems and methods

We require information from authors about some types of materials, experimental systems and methods used in many studies. Here, indicate whether each material, system or method listed is relevant to your study. If you are not sure if a list item applies to your research, read the appropriate section before selecting a response.

Materials & experimental systems

n/a	Involved in the study
☐	☒ Antibodies
☐	☒ Eukaryotic cell lines
☒	☐ Palaeontology and archaeology
☐	☒ Animals and other organisms
☒	☐ Clinical data
☒	☐ Dual use research of concern

Methods

n/a	Involved in the study
☒	☐ ChIP-seq
☐	☒ Flow cytometry
☒	☐ MRI-based neuroimaging

Antibodies

Antibodies used

For identification of human specific memory B cells for production of monoclonal antibodies, CD19-PE-Cy7 (PE-Cy™7 Mouse Anti-Human CD19, BD Pharmingen, cat. 557835, clone SJ25C1, lot. 8194923, 1:50 dilution), CD3-Pacific Blue (Pacific Blue™ Mouse Anti-Human CD3, BD Pharmingen, cat. 558117, clone UCHT1, lot. 8183535, 1:50 dilution), CD8-Pacific Blue (Pacific Blue™ Mouse Anti-Human CD8, BD Pharmingen, cat. 558207, clone RPA-T8, lot.8127596, 1:25 dilution), CD14-Pacific Blue (Pacific Blue™ Mouse Anti-Human CD14, BD Pharmingen, cat. 558121, clone M5E2, lot. 7164513, 1:50 dilution), CD27-APC-H7 (APC-H7 Mouse Anti-Human CD27, BD Pharmingen, cat. 560222, clone M-T271, lot. 8256900, 1:25 dilution), IgG-FITC (FITC Mouse Anti-Human IgG, BD Pharmingen, cat. 555786, clone G18-145, lot. 8284569, 1:12.5 dilution), IgM-PerCP-Cy5.5 (PerCP-Cy™5.5 Mouse Anti-Human IgM, BD Pharmingen, cat. 561285, clone G20-127, lot. 7278582, 1:50 dilution), IgD-PE-CF594 (PE-CF594 Mouse Anti-Human IgD, BD Horizon, cat. 562540, clone IA6-2, lot. 9114638, 1:25 dilution), anti-his-APC (Anti-6X His tag® antibody SureLight® Allophycocyanin, Abcam, cat. ab72579, clone AD1.1.10, lot. GR3192034-1, 1:25 dilution) and anti-his-PE (Anti-6X His tag® antibody Phycoerythrin, Abcam, cat. ab72467, clone AD1.1.10, lot. GR3223742-7, 1:25 dilution) antibodies were used.
For characterization of human antibodies, secondary anti-human IgG-HRP (HRP goat anti-human IgG (H+L) , ZSGB-BIO, cat. ZB-2304, polyclonal, lot. 118693, 1:5000 dilution), Anti-SARS-CoV-2 S2 mouse monoclonal Ab (MP biomedicals, Cat. 08720401, clone n.a., lot. S200414, 1:200 dilution), Anti-human IgG Fc secondary antibody PE (Biolegend, Cat. 410708, clone M1310G05, lot. B309947, 1:40 dilution), Goat anti-Mouse IgG (H+L) Cross-Absorbed Secondary Antibody, FITC (Thermo Fisher Scientific, Cat. A16073, polyclonal, lot. 42-123-062314, 1:200 dilution), and Anti-his antibody PE (Miltenyi Biotec, Cat. 130-120-787, clone GG11-8F3.5.1, lot. 5191227370, 1:200 dilution) antibodies were used.
For detection of SARS-CoV-2 infected cells, Rabbit anti-SARS-CoV-2 N protein monoclonal antibody (Abcam, Cat. ab281302, clone HL5511, lot. GR3395300-1, 1:1000 dilution) and Rabbit specific HRP polymer (Abcam, Cat. ab236469, clone n.a., lot. GR3388394-1, no dilution) antibodies used.

Validation

All the antibodies used in this study were commercial antibodies and were only used for applications, with validation procedures described on the following sites of the manufacturers:
CD19-PE-Cy7 (PE-Cy™7 Mouse Anti-Human CD19, BD Pharmingen, cat. 557835, clone SJ25C1, lot. 8194923, 1:50 dilution)
https://www.bdbiosciences.com/cn/applications/research/clinical-research/oncology-research/blood-cell-disorders/surface-markers/human/pe-cy7-mouse-anti-human-cd19-sj25c1-also-known-as-sj25-c1/p/557835
CD3-Pacific Blue (Pacific Blue™ Mouse Anti-Human CD3, BD Pharmingen, cat. 558117, clone UCHT1, lot. 8183535, 1:50 dilution)
https://www.bdbiosciences.com/cn/applications/research/t-cell-immunology/th-1-cells/surface-markers/human/pacific-blue-mouse-anti-human-cd3-ucht1-also-known-as-ucht-1-ucht-1/p/558117
CD8-Pacific Blue (Pacific Blue™ Mouse Anti-Human CD8, BD Pharmingen, cat. 558207, clone RPA-T8, lot.8127596, 1:25 dilution)
https://www.bdbiosciences.com/cn/reagents/research/antibodies-buffers/immunology-reagents/anti-human-antibodies/cell-surface-antigens/pacific-blue-mouse-anti-human-cd8-rpa-t8/p/558207
CD14-Pacific Blue (Pacific Blue™ Mouse Anti-Human CD14, BD Pharmingen, cat. 558121, clone M5E2, lot. 7164513, 1:50 dilution)
https://www.bdbiosciences.com/cn/applications/research/stem-cell-research/hematopoietic-stem-cell-markers/human/negative-markers/pacific-blue-mouse-anti-human-cd14-m5e2/p/558121
CD27-APC-H7 (APC-H7 Mouse Anti-Human CD27, BD Pharmingen, cat. 560222, clone M-T271, lot. 8256900, 1:25 dilution)
https://www.bdbiosciences.com/cn/applications/research/clinical-research/oncology-research/blood-cell-disorders/surface-markers/human/apc-h7-mouse-anti-human-cd27-m-t271/p/560222
IgG-FITC (FITC Mouse Anti-Human IgG, BD Pharmingen, cat. 555786, clone G18-145, lot. 8284569, 1:12.5 dilution)
https://www.bdbiosciences.com/cn/applications/research/b-cell-research/immunoglobulins/human/fitc-mouse-anti-human-igg-g18-145/p/555786
IgM-PerCP-Cy5.5 (PerCP-Cy™5.5 Mouse Anti-Human IgM, BD Pharmingen, cat. 561285, clone G20-127, lot. 7278582, 1:50 dilution)
https://www.bdbiosciences.com/zh-cn/products/reagents/flow-cytometry-reagents/research-reagents/single-color-antibodies-ruo/percp-cy-5-5-mouse-anti-human-igm.561285
IgD-PE-CF594 (PE-CF594 Mouse Anti-Human IgD, BD Horizon, cat. 562540, clone IA6-2, lot. 9114638, 1:25 dilution)
https://www.bdbiosciences.com/zh-cn/products/reagents/flow-cytometry-reagents/research-reagents/single-color-antibodies-ruo/pe-cf594-mouse-anti-human-igd.562540
anti-his-APC (Anti-6X His tag® antibody SureLight® Allophycocyanin, Abcam, cat. ab72579, clone AD1.1.10, lot. GR3192034-1, 1:25 dilution)
https://www.abcam.com/6x-his-tag-antibody-ad1110-surelight-allophycocyanin-ab72579.html
anti-his-PE (Anti-6X His tag® antibody Phycoerythrin, Abcam, cat. ab72467, clone AD1.1.10, lot. GR3223742-7, 1:25 dilution)
https://www.abcam.com/6x-his-tag-antibody-ad1110-phycoerythrin-ab72467.html
secondary anti-human IgG-HRP (HRP goat anti-human IgG (H+L) , ZSGB-BIO, cat. ZB-2304, polyclonal, lot. 118693, 1:5000 dilution)
http://www.zsbio.com/product/zb-2304
Anti-SARS-CoV-2 S2 mouse monoclonal Ab (MP biomedicals, Cat. 08720401, clone n.a., lot. S200414, 1:200 dilution)
https://www.mpbio.com/us/08720401-anti-coronavirus-spike-s2

Anti-human IgG Fc secondary antibody PE (Biolegend, Cat. 410708, clone M1310G05, lot. B309947, 1:40 dilution)
https://www.biolegend.com/en-us/products/pe-anti-human-igg-fc-11933
Goat anti-Mouse IgG (H+L) Cross-Absorbed Secondary Antibody, FITC (Thermo Fisher Scientific, Cat. A16073, polyclonal, lot. 42-123-062314, 1:200 dilution)
https://www.thermofisher.cn/cn/zh/antibody/product/Goat-anti-Mouse-IgG-H-L-Cross-Adsorbed-Secondary-Antibody-Polyclonal/A16073
Anti-his antibody PE (Miltenyi Biotec, Cat. 130-120-787, clone GG11-8F3.5.1, lot. 5191227370, 1:200 dilution)
https://www.miltenyibiotec.com/CN-en/products/his-antibody-gg11-8f3-5-1.html#pe:30-tests-in-60-ul
Rabbit anti-SARS-CoV-2 N protein monoclonal antibody (Abcam, Cat. ab281302, clone HL5511, lot. GR3395300-1, 1:1000 dilution)
https://www.abcam.cn/sars-cov-2-nucleocapsid-protein-antibody-hl5511-bsa-and-azide-free-ab281302.html
Rabbit specific HRP polymer (Abcam, Cat. ab236469, clone n.a., lot. GR3388394-1, no dilution)
https://www.abcam.cn/rabbit-specific-hrpdab-detection-ihc-detection-kit-micro-polymer-ab236469.html

Eukaryotic cell lines

Policy information about cell lines and Sex and Gender in Research

Cell line source(s)	The 293T cells and Vero E6 cells were obtained from ATCC. The 293F cells were purchased from Life Technologies. HeLa-hACE2 cells were kindly provided by Q. Ding at Center for Infectious Research of Tsinghua University. The A549 lung carcinoma cell line expressing human ACE2 were kindly provided by L. Wang from Duke-NUS Medical School.
Authentication	All cell lines were frequently checked for cellular morphologies, growth rates and functions.
Mycoplasma contamination	We confirmed that all cell lines were negative for mycoplasma contamination.
Commonly misidentified lines (See ICLAC register)	No commonly misidentified cell lines were used.

Animals and other research organisms

Policy information about studies involving animals; ARRIVE guidelines recommended for reporting animal research, and Sex and Gender in Research

Laboratory animals	Eight-week-old female K18-hACE2 transgenic mice (InVivos Ptd Ltd, Lim Chu Kang, Singapore) were utilized for this study. The housing conditions were 23±2 °C (High/Low temperature), 50±10% (High/Low humidity), and 12 h light and 12 h dark (Light cycle).
Wild animals	The study did not involve wild animals.
Reporting on sex	Sex was not considered in study design.
Field-collected samples	The study did not involve samples collected from the field.
Ethics oversight	Animal experiments were performed in a Biosafety Level 3 (BSL-3) facility in accordance with the National University of Singapore (NUS) Institutional Animal Care and Use Committee (IACUC) (protocol no. R20-0504), and the NUS Institutional Biosafety Committee (IBC) and NUS Medicine BSL-3 Biosafety Committee (BBC) approved SOPs.

Note that full information on the approval of the study protocol must also be provided in the manuscript.

Flow Cytometry

Plots

Confirm that:

☒ The axis labels state the marker and fluorochrome used (e.g. CD4-FITC).

☒ The axis scales are clearly visible. Include numbers along axes only for bottom left plot of group (a 'group' is an analysis of identical markers).

☒ All plots are contour plots with outliers or pseudocolor plots.

☒ A numerical value for number of cells or percentage (with statistics) is provided.

Methodology

Sample preparation	PBMCs from convalescent individuals were collected and incubated with an antibody and recombinant spike trimer cocktail for identification of spike-specific B cells. The cocktail consisted of CD19-PE-Cy7, CD3-Pacific Blue, CD8-Pacific Blue, CD14-Pacific Blue, CD27-APC-H7, IgG-FITC (or IgM-PerCP-Cy5.5, IgD-PE-CF594) (BD Biosciences) and recombinant wild-type spike-Strep or spike-His purified in our laboratory. Three consecutive staining steps were conducted. The first was using a LIVE/DEAD Fixable Dead Cell Stain Kit (Invitrogen) to exclude the dead cells. The second was mixing with an antibody and recombinant spike cocktail to identify spike-specific B cells. The third was to target the recombinant spike trimer captured on the surface of B cells by either Streptavidin-APC (eBioscience) or anti-his-APC/PE antibodies (Abcam). The stained cells were thoroughly washed and resuspended in PBS before strained through a 70 µm cell mesh (BD Biosciences). More information were available in Methods sections.

Instrument	BD Aria II
Software	FlowJo version 10
Cell population abundance	The spike-specific B cells constitute about 0.16%-4.90% among the CD27+IgG+ or IgM-IgD- B cell population. More Information were available in Extended Data Fig.1.
Gating strategy	The spike-specific B cells were gated as CD19+CD3-CD8-CD14-CD27+IgG+spike+ or CD19+CD3-CD8-CD14-IgM-IgD-spike+. More Information were available in Extended Data Fig.1 and Methods sections.

☒ Tick this box to confirm that a figure exemplifying the gating strategy is provided in the Supplementary Information.
